# Political and socioeconomic factors that shaped health taxes implementation in Peru

**DOI:** 10.1136/bmjgh-2023-012024

**Published:** 2023-10-09

**Authors:** Mario Zuleta, Silvana Perez-Leon, Melissa Mialon, Jaime Delgado-Zegarra

**Affiliations:** 1 Centre for Food Policy, City University, London, UK; 2 CRONICAS Center of Excellence in Chronic Disease, Universidad Peruana Cayetano Heredia, Lima, Peru; 3 Trinity Business School, Trinity College Dublin, Dublin, Ireland; 4 Facultad de Ciencias Administrativas y Recursos Humanos, USMP, Lima, Peru

**Keywords:** health policy, public health, qualitative study

## Abstract

**Background:**

In 2016 and 2018, the Peruvian Ministry of Economy and Finance (MoEF) significantly reformulated taxes on tobacco products, alcohol and sugar-sweetened beverages (SSBs). During these processes, different actors advanced arguments supporting or opposing the taxes. This study examines Peru’s political and socioeconomic factors, the role of other actors and framing strategies, shaping health taxes introduction.

**Methods:**

We conducted qualitative analysis by collecting information from three sources, such as: (1) media material (n=343 documents), (2) government documents (n=34) and (3) semistructured interviews (n=11). That data allowed us to identify and characterise the actors involved in implementing health taxes in Peru. We combined the data from these sources, synthesised our findings and conducted a stakeholder analysis.

**Results:**

Key actors supporting taxes were the MoEF and civil society organisations, while trade associations and the alcohol, SSBs and tobacco industries opposed them using economic, trade-related arguments and criticised the policy process. The supporting group used arguments related to the economy and health to legitimate its narrative. The framing strategies employed by these stakeholders shaped and determined the outcome of the policy process.

**Conclusion:**

Peruvian stakeholders against health taxes demonstrated a strong capacity to convey their messages to the media and high-level policy-makers. Despite these efforts, attempts to interfere with health taxes were unsuccessful in 2016 and 2018 and failed to overcome state institutions, particularly the MoEF. Strong institutions and individual decision-makers in Peru also contributed to the successful implementation of health taxes in Peru in 2016 and 2018.

**WHAT IS ALREADY KNOWN ON THIS TOPIC**

- There is extensive literature worldwide on the political processes that affect the implementation of public health policies, particularly health taxes. Areas such as the effectiveness of implementation, the stakeholders’ participation and industry interference have been more widely addressed by studies.
- In the case of Peru, the implementation of health taxes has been more focused on tobacco than other damaging products Nevertheless, since sugar-sweetened beverages tax adjustments in 2018 more literature regarding these products has started to be produced.

**WHAT THIS STUDY ADDS**

- This is the first time that health taxes in Peru have been analysed using a political lens, which comprehends the sociopolitical factors that led to the implementation of health taxes policies.
- This study used a framing analysis to understand the arguments of both stakeholders in favour and against health taxes and identify the main stakeholders stating these positions, which had not been studied before in Peru.

**HOW THIS STUDY MIGHT AFFECT RESEARCH, PRACTICE OR POLICY**

- This study offers important insights which can demonstrate and illuminate how political processes affect the implementation of public policies in countries such as Peru.
- The results from our study, which focused on the successful adoption of health taxes, may inform other countries currently discussing similar public policies.
- Gathering public health and economic-related evidence about the impact of health taxes is an excellent way to confront narratives and arguments against these taxes.

## Background

There is a strong evidence that consuming products such as alcohol, sugar-sweetened beverages (SSBs) and tobacco contribute to the increase in non-communicable diseases (NCDs).^1 2^ The annual costs to Peru’s public health system associated with NCDs resulting from consuming harmful products exceed US$3 billion and constitute almost half of the public health budget.^3^ This leads countries to implement public policies to reduce and discourage the consumption of these harmful commodities. Health taxes are considered cost-effective and, as such, one of the WHO’s ‘Best Buys’ interventions. Health taxes have better results when applied together with other interventions such as advertising restrictions, labelling regulations and education campaigns.^4 5^

As of 2021, 21 countries in the Region of the Americas have taxes on SSBs,^5 6^ and 21 countries have also signed the WHO Framework Convention on Tobacco Control (WHO FCTC).^7^ Moreover, 29 countries have an alcohol excise tax on beer, wine and spirits.^8^ Among the countries that have implemented taxes on alcohol, SSBs and tobacco, Peru has achieved some progress. Discussions on health taxes generated a public debate, particularly in 2016 and 2018, with the participation of different actors from the public and private sectors. In 2016 and 2018, the Ministry of Economy and Finance (MoEF) made adjustments to a tax called Impuesto Selectivo al Consumo (Selective Consumption Tax), originally intended to discourage the consumption of products with negative ‘externalities’. In 2016, the main discussion was on the increase of taxes on tobacco. In 2018, the debate was around the introduction of taxes on SSBs, and there were also important readjustments to taxes on alcoholic beverages at that time.

There is still a limited understanding of the factors that lead to adopting these health taxes: the political strategies adopted by supporters and opponents of the taxes and the discourse and arguments they put forth.^9^ There is a gap that needs to be filled with evidence on the underlying factors that have led to the adoption of health taxes in Peru and the lessons to be learnt, which could guide other countries undergoing a similar process. Our study, therefore, focused on the political and socioeconomic factors that shaped the implementation of these health taxes in Peru, particularly the role of different actors, their arguments and framing strategies.

## Methods

We used qualitative methods, including a review of scientific studies; an analysis of media material from 2016 to 2018; a review of documents from the Peruvian government; and 11 semistructured interviews. The study was undertaken between February 2022 and July 2022. Data were in Spanish and English.

### Review of media material and government documents

To investigate the framing of health taxes in Peru, we conducted a systematic analysis of media coverage, building on similar studies.^10 11^ There are two databases that include written news articles for Peru: LexisNexis and Factiva. While Factiva provides access to a broader range of years and more written news media, we only had access to this database and not LexisNexis. We retrieved data on health taxes from Factiva’s ‘Global News Monitoring and Search Engine,’ including news articles, opinion pieces, interviews and press releases published in both English and Spanish.

To supplement these data, we also conducted a manual search of news and other documents, guided by recommendations from key informants with expertise in health taxes in Peru (see Systematic search strategy).

#### Systematic search strategy

**Figure 1:**
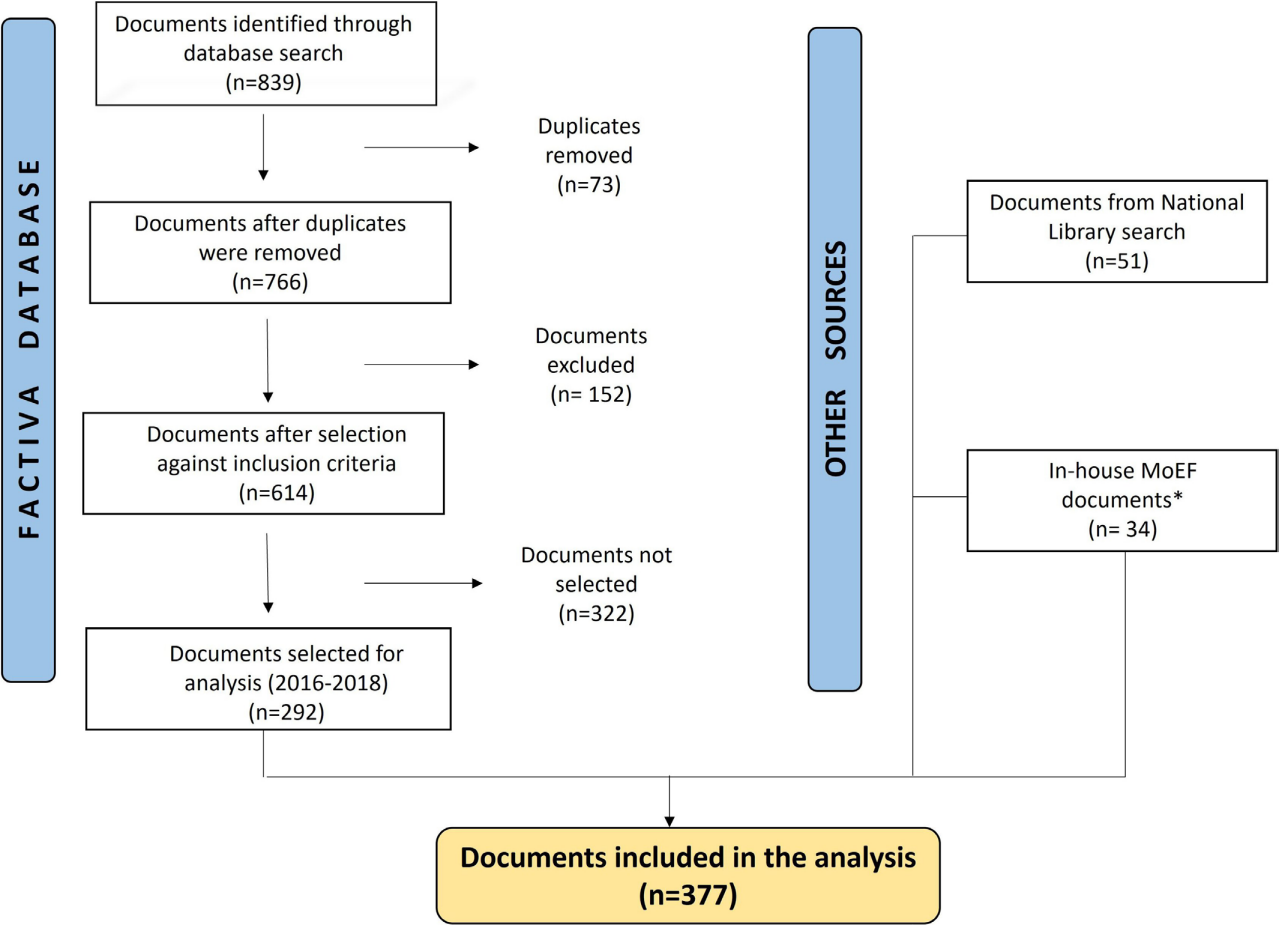
Flow chart doc analysis. *Documents accessed through transparency law Na27806. MoEF, Ministry of Economy and Finance.

We searched documents discussing the selective consumption tax, where at least one of the products under the Peruvian health taxes—alcohol, tobacco or SSBs—was discussed (figure 1). Details of the search strategy are available in online supplemental file 1.

10.1136/bmjgh-2023-012024.supp1 Supplementary data

#### Eligibility criteria

After removing duplicates, all documents were reviewed by at least two researchers from a group of three: two researchers (MAZ and SP-L) and one research assistant. This was an iterative reflective process where non-agreements were always discussed among the team. Documents were included if they mentioned health regarding any of the three products to be analysed. They were excluded if they referred to health taxes on other commodities (crude oil, automobiles) or were merely a translation of other material already included. Figure 1 is a flow chart that details the inclusion or exclusion of documents through that process.

We reviewed media documents from 1999 to the time of data collection and identified two key periods as the most relevant for our study, with a drastic increase in the production of news on health taxes: in 2016 with more news discussing tobacco and in 2018, with information more focused on SSBs. We further analysed documents from these years only (292 documents).

#### Other media material

We searched for news articles through the National Library’s depository, including the most-read newspaper, ‘*Trom*e’ (not available on Factiva), and the economic newspaper ‘*Gestión*’, only covering May 2018, when health taxes were widely discussed. From this search, we found 51 articles.

#### Documents from the Peruvian government

We also accessed other documents identified by key informants (see below). We accessed 34 documents from the MoEF through the ‘Transparency and Access to Public Information Law—No 28706’.^12^ These documents, which showed the exchanges between government and industry, were important to complement and contrast the limited information we got from other sources for these stakeholders.

#### Analysis of media and government material

The process of inclusion/exclusion of documents was a first step to extract some initial relevant data: media source, date of publication, language, title, type of news (information, interview, op-eds, etc), stakeholders mentioned, and its stance on health taxation (see online supplemental file 2). That process was informed from our revision of the literature, our expertise on the topic and additional discussions within the team, until agreement was reached. A preliminary codebook for thematic analysis was prepared, with two groups of codes: arguments supporting and arguments against health taxes. The codebook (see online supplemental file 3) was later used to code the selected 292 documents using ATLAS.ti (V.8.4.26.0). Our analysis was of a qualitative nature, so we did not quantify the arguments into each group (supporting or against health taxes). In the manuscript, we present the arguments within each of these groups.

### Semistructured interviews

The media analysis and document review allowed us to identify key stakeholders involved in the discussion of 2016 and 2018 around health taxes. We contacted them and used the snowball sampling method^13 14^ to get other possible interviewees. Different stakeholders were reached so we could cover different opinions from various sectors such as the media, the government, for example, Ministry of Health (MoH) and MoEF, public opinion leaders, researchers, advocates working for civil society organisations, think tanks, representatives from companies and trade associations. In total, we reached 26 individuals, of which 15 did not respond or declined due to difficulties in scheduling an interview. Between April and July 2022, we conducted 11 interviews (3 civil society representatives, 3 public opinion leaders, 2 government representatives, 1 researcher, 1 industry representative and 1 international organisation representative). We reached saturation, in terms of the topic discussed with our interviewees from academia and civil society, and we could not get any more interviews from the government or the industry due to time constraints.

The interview guide was developed (see online supplemental file 4) by two researchers (MAZ and SP-L) with extensive knowledge of the local context, and later reviewed by the rest of the team. All interviews were conducted online by MAZ and SP-L using the Zoom platform and lasted between 45 and 90 min. An informed consent form was orally approved. Interviews were audio recorded with the consent of our informants, and notes were taken to help with the analysis.

All interviews were transcribed verbatim by researcher MAZ and a professional transcriber, who also worked under the ethics consent rules of our study.

### Stakeholders analysis

First, for the stakeholders analysis,^15 16^ we identified key individuals by searching the Factiva database. In this process, two researchers (MAZ and SP-L) independently analysed which arguments were in favour and against taxes and discussed the stance of these stakeholders. Second, we asked our interviewees whom they would consider relevant stakeholders, which allowed us to confirm or expand our initial list of stakeholders.

### Reporting

Here, we describe the context for the Peruvian health taxes in more detail, based on the above analysis conducted. We then present the arguments of those in favour and those against health taxes before introducing our findings from our stakeholder analysis. We refer to online supplemental file 5 when presenting quotations from our interviews.

### Patient and public involvement

Neither patients nor the public were involved in any stage of the realisation of this research.

## Results

### Peruvian context

Prior to the 1990s, Peru was subject to government protectionism and interventions on prices. There were also restrictions on imports and a growing external debt, among other measures that resulted in a hyperinflation and a recession.^17^ During the 1990s, Peru underwent economic liberalisation in the context of that financial crisis. The shift from the 1990s implied adopting policies dictated by the International Monetary Fund (IMF) and other international organisations aimed at stabilising the economy. In this context, the Selective Consumption Tax, which specifically taxed products considered to be luxury goods such as fuel, tobacco products, new vehicles, carbonated soda and alcoholic beverages, was introduced in 1999.^18^ This tax was raised to different levels according to each product category. These changes, as well as the tax policy more generally, were designed and implemented by the MoEF. In Peru, unlike many other countries, changes in health taxes rates do not go through Congress but are modified solely by the MoEF.

The political contexts allowed the health taxes adjustments in 2016 and 2018. In 2016, the adjustments to health taxes came at the end of the then government in place (which lasted from 2011 to 2016). However, in 2018, changes came at the start of a new government after the resignation of the former President of the Republic. MoEF officials interviewed for our study considered these periods windows of opportunity for tax adjustments (online supplemental material 5, cite 1).

In May 2016, the MoEF raised cigarette taxes by 157%, the most significant increase in Peru’s history of health taxes.^19^ This was the first time in 6 years (since 2010) that cigarette taxes were changed. The adjustment was widely debated and generated strong criticism from opposing stakeholders. Interviewees reported that one of the reasons for this slow process was intense lobbying by the tobacco industry in Congress which, although not deciding directly on the taxes, seemed to be a venue for influence by the industry (online supplemental material 5, cite 2).

In May 2018, several taxes were adjusted, including beverages with more than 20% in alcohol concentration, where taxes were raised from 25% to 40%, and cigarettes taxes which were increased by 50%. SSBs containing more than 6 g/100 mL of sugar were subjected to a 25% tax^20^ while other beverages maintained a 17% tax rate. This increase was considered a turning point since it was the first time SSBs were taxed based on their sugar levels. Despite being modified in 2018, alcohol taxes did not receive much attention in the policy agenda as SSB taxes dominated public discussion. While there was a debate on how beer taxes should be levied in 2014–2015, we did not analyse it as it was outside the scope of our study. Similarly, gas taxes were an important topic in 2018 but not analysed in our study due to their specific nature and changing policy dynamics.

The President of the Republic and the Minister of Economy both supported these taxes, and their opinions were often highlighted in the media, which placed the issue of health taxes on the public agenda. In the following months of May 2018, a dispute occurred between the President and the MoEF related to a strike threat from the transporter’s union due to increased health taxes on gas. The President soon gave signs that he might not be so supportive of taxes, for political reasons. In this context, the Minister of Economy lose political support, which led him resigning from his position.^21^

Despite the country’s political instability and institutional crisis in recent years, which has led to six changes of Presidents between 2016 and 2022, the MoEF is considered a stable government institution, with its officials in charge of taxation, most of which took office in 2014 and remained in their positions in the institution at least until the period of data collection for this study.

In the following section, we present the arguments in favour and those opposing health taxes, as identified through our analysis. The following table 1 summarises the main arguments that will be outlined in more detail in the section below.

**Table 1:** Arguments supporting and against health taxes

Arguments supporting health taxes	Health-related arguments	Reduce consumption of harmful commodities
		Compensate negative externalities
		Impact of harmful commodities on public health
	Economic-related arguments	Source of revenue
		Health taxes would not have a negative economic impact
		Increase labour productivity
	Arguments from international supporters and experiences	International organisation’s statements in favour of health taxes
		Comparison with other countries
Arguments against health taxes	Economic-related arguments	Impact on prices, sales, employment and investments
		Most affected poor sectors, small businesses and women
		Jobs would be put at risk
	Illegal commerce-related arguments	Smuggling increase
		Tax evasion increase
		More product adulteration
	Process-related arguments	Lack of dialogue with the industry
		Volatility and unpredictability
		Lack of transparency

### Arguments supporting health taxes

There was a broad range of arguments supporting health taxes. Those considered most relevant were health-related arguments, economic-related arguments and arguments that international organisations and the experiences of other countries stated.

### Health-related arguments

Some common arguments for advocating for health taxes were related to the potential health benefits that would happen if the taxes were introduced. These arguments were, in most cases, advanced by the MoEF as one of the main actors advocating for health taxes. The MoH also expressed its support for taxes and used similar arguments related to health, but less frequently than the MoEF.

We identified three main health arguments, such as: (1) health taxes would reduce the consumption of harmful commodities; (2) health taxes would compensate for the negative externalities caused by harmful commodities and (3) harmful commodities impact negatively on public health.

The argument (1) that health taxes would reduce the consumption of harmful commodities was used in 2016 to refer to the consumption of tobacco products, specifically in children and teenagers, an argument supported by Pan American Health Organization (PAHO/WHO) and the World Bank.^22 23^ In 2018, this argument was used in the context of introducing the SSB taxes concerning the above positive experience in the 2016 tobacco taxes increase.

About the argument of the (2) negative externalities, the MoEF pointed out the negative consequences of consuming harmful products using evidence from abroad.^24 25^ Our interviewees from the MoEF noted that the concept began to be used internally in official documents between 2005–2006, long before the taxes were introduced (online supplemental material 5, cite 3).

And more or less in the year 2005–2006, this concept began to appear, it is already mentioned that selective consumption taxes (health taxes) are applied on goods and services that cause negative externalities, so the concept of negative externalities begins to appear in the guidelines (MoEF official).

However, in our review of the media, we found evidence for the concept to be used publicly just in 2016 and more widely in 2018, when the discussion of health taxes was on the public agenda.

One argument widely used by those advocating for health taxes was referred to (3) the negative impacts of harmful commodities on public health, especially mentioning diseases, such as cancers in the case of tobacco smoking in double and obesity, in the context of the consumption of SSBs. These arguments were, in some cases, supported by epidemiological data about the incidence of diseases. The direct and indirect costs of diseases such as cancer, obesity or diabetes for the government were also mentioned,^26 27^ with health taxes being seen as a solution to mitigate these indirect costs.

#### Economic-related arguments

Those advocating for health taxes used arguments related to the economy, noting the double dividend on the economy and population health if the taxes were implemented.

Government officials, journalists/columnists, public opinion leaders and, in fewer cases, civil society organisations used these arguments. Something particular about these arguments was that they were commonly used by high-ranking government members, such as the President of the Republic and the Ministry of Economy, as noted earlier. Using an economic argument from these authorities may have attracted media attention. It could have responded to the arguments from the industries and trade associations against health taxes, which also used economic arguments—which we will discuss further in the present manuscript.

Two leading economic arguments were identified supporting health taxes: (1) health taxes would be a source of revenue for the government and (2) health taxes would not have a negative economic impact. In the first argument, the MoEF publicly stated the need to ‘make cash’.^28^

The Minister of Economy, David Tuesta, defended the recent increase in the Selective Consumption Tax (ISC) rates for some products and acknowledged that this decision was based, in part, on the actions required to ‘make cash’ in addition to correcting negative externalities. (05/15/2018, Gestion newspapaer)

Still, every time government representatives, even the President, used these arguments in favour of health taxes, they also mentioned health-related statements, which might be considered a validation strategy.^29 30^

The framing of health taxes as a source of revenue had its critics, especially industry-related stakeholders and public opinion leaders, against health taxes. For them, the real purpose of the taxes was generating income,^31^ not improving the population’s health. They stated that taxes were the ‘easiest way’^29^ for the government to obtain revenue, that it was ‘perverse’^32^ as it would benefit from the consumption of harmful products, and even that taxes were a ‘cock-and-bull story of cancer and glucose’^33^ that took advantage of the products ‘bad press’.^34^

In their second argument, current and former government officials pointed out that the economic impacts of the taxes would be ‘minimal’^35^ or ‘not significant’,^36^ which seemed to be a response to potential adverse reactions of the public opinion, and concerns of those who opposed the taxes and predicted price increases, among other negative impacts. In addition, government actors mentioned that increases in health taxes would, on the contrary, result in a positive effect on the economy due to the rise in labour productivity due to a healthier population. When this was mentioned, it was commonly supported by data on the gross domestic product or the public costs of NCDs.^31 35–37^

#### Arguments from international supporters and experiences

The third group of arguments used to advocate for health taxes was arguments showing the support from international organisations to legitimate its importance. First, opinions and statements from international organisations were highlighted, such as those of the WHO, including the WHO FCTC, as a leading treaty, primarily when tobacco taxes increased in 2016, and those of the World Bank.^22 23^ This international support—as an argument in favour—was also used while comparing Peru with other countries, such as Mexico, whose experience in increasing health taxes was often mentioned to legitimise the increase or the inclusion of new products, such as SSBs, in 2018.^37–39^ A revealing contrast is that while officials from international organisations such as the Organisation for Economic Co-operation and Development (OECD), PAHO and the IMF congratulated Peru for its implementation of health taxes,^40–42^ representatives from the soft drinks industry and the exporter’s association (COMEX) claimed that health taxes would affect international agreements Peru had signed.^43–45^

### Arguments against health taxes

For those opposing health taxes in Peru, there were three more frequently used arguments: those related to the economy, those related to illegal sales and other arguments related to the process of health taxes adoption and implementation.

#### Economic-related arguments

Most of the arguments against health taxes had an economic background behind them, used mainly by industry-related actors. The most common concerns were that health taxes would impact prices and sales, decreasing employment and investments and negatively impacting market competition.^43 46–48^ The Association of Minimarket Salespersons (ABP) prominently used these messages.^49–51^

Industry representatives and business associations stated that the weakest parts of the value chain and the sectors with lower incomes would be the most affected after taxes implementation.^52 53^ It was pointed out, for example, that the taxes would affect small businesses ‘more than 300 000 minimarkets’ (sometimes this number was over 400 000),^49 54^ most of them run by women,^55^ or that about ‘20 000 workers and transporters’ would be affected and that their jobs would be put at risk.

#### Illegal commerce-related arguments

Another group of arguments widely mentioned by opposing stakeholders focused on the detrimental consequences on the trade of these products, either by showing that adverse outcomes have occurred after their implementation or by anticipating what would happen in the future.

The leading trade associations stated that increasing health taxes would increase informality and illegal activities such as smuggling and production of adulterated products.^51 56–59^ The industry-funded observational studies, undertaken by consulting firms, were considered ‘unreliable’ by MoEF officials (online supplemental material 5, cite 4), as they lacked a methodology and were merely presentations slides summarising the results (we could not access these studies for this study to assess their strength):

Every time we have a modification, they come up with studies, with information, which in reality are not very reliable but which they can afford to finance and have public exposure of these reports (MoEF official).

The beer and tobacco industries highlighted the risk that tax evasion would occur after smuggling increased, the amount the government would lose in revenues. They emphasised the gap that would open up between formal and informal sales and called for more state control over smuggling.

Associations’ representatives and public opinion leaders who criticised the health taxes adjustments stated that the alleged lack of concern from the government about smuggling demonstrated that they ‘do not know the reality of the country’^56^ and were ‘turning its back on reality’.^57^

Similarly, opponents of health taxes argued that products would be adulterated. Trade association representatives and public opinion leaders said that these products would even put people’s health at risk,^60^ creating ‘a market for unsafe and sugary homemade drinks’.^61^ The brewing industry pointed out that this consumption was ‘the most harmful form of alcohol consumption for people’s health’.^53^

#### Process-related arguments

The third set of arguments against health taxes was questioning the process of their adoption and implementation. Industry representatives claimed that there was a lack of dialogue and little discussion with the industries; and criticised the alleged volatility or unpredictability of the measure. The industries also sought to emphasise that they were willing to talk to the government but had not been heard and considered that the taxes adjustments were ‘arbitrarily decided’.^54^

Trade associations also referred to the lack of ‘transparency and planification’ of the policy. They argued that the ‘rapid increase’ of taxes was ‘surprising’ and that they did not have a reasonable time frame to implement it.

### Stakeholder analysis

The above messages reflected the concerns and interests of different stakeholders. The stakeholder analysis (table 2) helped identifying the actors and their positions, supporting or opposing health taxes. We found that there were a more significant number of actors supporting health taxes than those who opposed them. Those with the most media coverage supporting health taxes were government officials from MoEF, civil society organisations and public health experts. In most cases, the actors fighting health taxes were trade associations and industries.

**Table 2:** Health taxes stakeholders in Peru 2016–2018

Type of organisation		Health taxes position*
Trade associations	National Society of Industries	Against
	Association of Soft Drinks and Non-alcoholic Beverages	Against
	Exporters Association	Against
	Chamber of Commerce of Lima	Against
	American Chamber of Commerce of Peru	Against
Tobacco industry	British American Tobacco	Against
Alcohol industry	Backus Beer Company	Against
Civil society organisations	National Federation of Food, Beverage and Allied Workers’ Federation	Against
	Public opinion leaders†	Against/in favour/neutral
	National Anti-Tobacco Commission	In favour
	Centre for Information and Education for Drug Abuse Prevention	In favour
	Association of Minimarket Salespersons	Against
International organisations	Pan American Health Organization	In favour
	WHO	In favour
	World Bank	In favour
	Organisation for Economic Co-operation and Development	In favour
	International Monetary Fund	In favour
Government	Ministry of Economy and Finance	In favour
	Directorate General for Public Revenue Policy	In favour
	Ministry of Health	In favour
	National Tax Agency	In favour
	Health Commission of Congress	Against
Academy/think tanks	Development Analysis Group	In favour
	Institute of Peruvian Studies	In favour

*Two researchers (MAZ, SP-L) independently discussed the stance of stakeholders (in favour, against or neutral).

†In favour: Elmer Huerta, Patricia Ritter, Waldo Mendoza, Oscar Ugarte, Hugo Santamaría, Alonso Segura; Against: Aldo Mariátegui, Mario Zúñiga, Sandro Fuentes, Anibal Quiroga, Oscar Súmar; Neutral: Luis Minaya, Pablo Sotomayor, Elmer Cuba, Alfredo Torres.

Except for Peru’s major beer company, it was rare that companies themselves came out in the discussion on health taxes. The tobacco industry played a minor role publicly; its low profile in Peru may be due to higher public scrutiny than other sectors.^62^ Meanwhile, SSB companies publicly had a more prominent role, through their trade association, especially in the 2018 taxes increase. Trade associations were important actors opposing health taxes. These represented companies involved in alcohol, tobacco and SSBs, besides other industries. The SSB companies (such as Coca-Cola Peru and PepsiCo) were represented by the Association of Soft Drinks and Non-alcoholic Beverages. The alcohol and tobacco industries were represented by the National Society of Industries, but also others such as the National Confederation of Private Businesses (Confiep), the Exporters Association (COMEX) and the Chamber of Commerce of Lima. These actors were the ones with higher media exposure in the media. Competing companies collectively used trade associations to express common positions and opinions. Another vital stakeholder was the ABP, portrayed as a civil society organisation with close industry links. This organisation was especially active in expressing its opinion against health taxes and had high exposure in the media. Through our interviews, we found that not only these actors had a strong media presence in 2016 and 2018, but they were also active in their attempts to approach political actors in Congress to try having legislative measures introduced, not only related to taxes but also others that they considered being economically damaging to the industries (online supplemental material 5, cite 5).

In turn, a leading actor in the political discussion championing health taxes was the MoEF. Key informants belonging to civil society, international organisations, academia and the government referred to the MoEF as an institution whose stability and institutionalism was crucial for advancing the adoption and adjustments to health taxes, especially from officials in charge of taxation, who combined both the arguments of economic efficiency with public health purposes (online supplemental material 5, cite 6).

So there was a debate in Congress and if you listen to the representative of the Ministry of Economy, you would think that she is a person who represents health, I would say that she has presented better than any representative of the Ministry of Health. (Civil society health tax advocator).

On the other hand, actors opposing health taxes criticised the MoEF, saying it was a ‘biased’, ‘ideological’ institution (online supplemental material 5, cite 7) that did not want to listen to the private sector (online supplemental material 5, cite 8).

International organisations such as the WHO, the PAHO (PAHO/Office of the WHO of the Americas), the World Bank, the IMF and the OECD were also essential stakeholders that made public statements (online supplemental material 5, cites 9 and 10) advocating for health taxes in 2016 and 2018.^27 41 46^ Through the interviews with MoEF’s officials and international agencies, it was noted that there was a fluid dialogue between these institutions as they provided technical recommendations for health taxes policies.

Other stakeholders in favour of the health taxes were civil society organisations such as the National Anti-Tobacco Commission, and the Centre for Information and Education for Drug Abuse Prevention, academic think tanks such as the Development Analysis Group, the Institute of Peruvian Studies, and independent academics and public health advocators. These institutions and individuals provided evidence and expert opinions.

On the other hand, the MoH did not have a leading role in the health taxes discussion but was instead consulted to provide health data for establishing health taxes. Interviewees from the MoEF and MoH considered the latter to be a fragmented institution, understaffed, with limited staff continuity, and, therefore, a weak capacity to plan long-term public policies. This role was, in some cases, questioned in the media, as these policies related to health did not come from the MoEF (online supplemental material 5, cite 11).

The Health Commission of Congress was another institution where health taxes and public health policies faced significant opposition. According to some of our interviewees, attempts to introduce new public policies were stopped due to industry lobbying. Obstructionist strategies and actions such as delaying or not scheduling legislation related to regulating harmful commodities were reported practices. A remarkable event occurred in 2016^63^ when, shortly before voting on the law banning tobacco advertising, promotion and sponsorship, the then President of the Health Commission decided to withdraw from the session for no apparent reason, and the discussion could not be carried out (online supplemental material 5, cite 12). Later, it was made public that the political party to which that congressman was part received unaccounted funding from trade associations to which the major tobacco company belonged.^64^

More recently, in 2021, there were attempts in Congress from different political parties to pass laws^65 66^ to remove the power from the MoEF to readjust taxes, giving that power to Congress instead. However, those attempts were unsuccessful.^67^

## Discussion

The independence of the MoEF from the Congress to adjust taxes is unique to Peru, compared with other countries in the region.^68^ This means that there was not as much political debate and lobbying around the introduction and changes on health taxes in Congress. This autonomy of the MoEF, together with the stability of that institution, despite changes in leadership in the government, can be considered key factors that allowed the effective adoption and implementation of health taxes. Unlike other political processes in other countries where interministerial support was an important factor,^68–70^ the MoEF is, therefore, a leading actor for health taxes in Peru.^71^ In contrast, other ministries such as the MoH played a marginal role due to institutional weaknesses. Civil society engagement and reference to international agencies were also crucial in Peru, which is congruent with the literature.^69 72^ Peru’s alignment with a global public health agenda and the WHO FCTC has been beneficial for implementing health taxes and getting external support. The recurrent mention of ‘early adopter’ countries^71^ within the region, and with successful adoption of health taxes and similar sociopolitical backgrounds^68 73 74^ were also essential for legitimising the need for such public policy.^68^ More research on these processes could deepen whether, in these cases, we could be talking about policy transfer or policy diffusion networks.^71 75 76^

The use of trade association to represent companies in the alcohol, SSBs and tobacco industries was also noted in other studies.^68 72 77^ This showed the unity and strength of these sectors and made their positions readily accessible to the media and the government. This coalition-building strategy through trade associations serves as a protection where the companies are less visible, but can combine their resources, and experience in public policy agenda setting.

Similarly, the industries also employed other groups to legitimise their arguments and try to influence agenda-setting.^78^ This was seen with the ABP, which has links to the tobacco industry and was identified as one of the most active actors with a strong media presence and ties with political parties in Congress.

Economic considerations were identified as central arguments, both in favour and against health taxes. When actors supporting health taxes, especially those from the government, gave economic arguments, they were primarily accompanied by health-related statements. This framing may be considered a validation strategy legitimising health taxes and increasing public support for them, as has been pointed out in specific cases, such as taxes on SSBs in other countries.^68 79^ The argument advocating for health taxes as a source of revenue in Peru differs from that found in other studies.^71^ Trade associations mentioned that health taxes would increase job losses, prices and informality, among other economic concerns, an argument also found elsewhere.^62^ The industries also argued against health taxes for their alleged regressivity, highlighting that those most affected were actors in the supply chain, an argument also found in other countries but not backed up by scientific evidence.^69 71 74 80^ The criticism of the policy process from the industries is rarely reported in the literature on health taxes. These arguments revealed a concern for how health taxes were adopted, even when good governance practices were observed in these processes.

### Strengths and limitations

Applying a systematic media search process allowed us to analyse a longer time frame before focusing on more precise moments. Also, deductive scope during systematic media analysis allowed us to explore a broader range of given arguments. Therefore, we believe in having captured all relevant statements from stakeholders.

Some limitations should be noted. First, we only included three products in our analysis, and other arguments may have been used in Peru products such as fuels. We had difficulties accessing more interviewees from sectors of government involved with health taxation. We also had more information from industry-related sources in the case of SSBs, which may have biased our overall findings for this sector.

## Conclusion

As in several contexts where the implementation of taxes encounters strong obstacles, mainly from industry and its allies, stakeholders also demonstrated a strong capacity to convey their messages to the media and high levels of decision-making. Although attempts to interfere with health taxes policies were unsuccessful in 2016 and 2018 and failed to overcome state institutions that implemented health taxes and received support from other civil society actors.

Institutional factors and political context contributed to the successful implementation of health taxes in both years. We found that the committed participation and stability of government actors, particularly that of the MoEF, despite a chaotic last few years and continuous changes in the Republic’s Presidency, were fundamental for the adoption of health taxation. However, this reality does not exempt itself from an inherent fragility in volatile political contexts such as Peru. The sustainability and progress of such public health measures require empowered state institutions with sufficient strength and independence to stand up to the industry’s influence. For that purpose, it is necessary to encourage the production of local evidence from different framings, not only health related, to accompany policy-makers’ decisions to confront the narratives and arguments against health taxes.

## Data Availability

All data relevant to the study are included in the article or uploaded as online supplemental information.
