# Supplementary material for: Political and socioeconomic factors that shaped health taxes implementation in Peru
Source: BMJ Glob Health. 2023 Oct 9;8(Suppl 8):e012024. doi: 10.1136/bmjgh-2023-012024 (PMC10565308; doi:10.1136/bmjgh-2023-012024)
Supplement: Supplementary data [file bmjgh-2023-012024supp001.pdf]

# Supplementary material

Supplementary material 1: Search terms used for the Factiva database

| | |
|---|---|
| **Text** | (ISC OR Impuesto Selectivo al Consumo) AND (bebida* azucarada* OR gaseosa* OR alcohol* OR cerveza* OR cigarr* OR tabaco OR beverage* OR sugary OR soda* OR "soft drink*" OR beer* OR tobacco) |
| **Date** | All Dates |
| **Source** | All Sources |
| **Author** | All Authors |
| **Company** | All Companies |
| **Subject** | All Subjects |
| **Industry** | All Industries |
| **Region** | Peru |
| **Language** | All Languages |
| **Results Found** | 839 |
| **Timestamp** | 1990 - 11 February 2022, 17:35 |



Zuleta M, *et al. BMJ Glob Health* 2023; 8:e012024. doi: 10.1136/bmjgh-2023-012024

Supplementary Material 2: Media material data

| Media Source | Date | Language | Title | Type of news (Informative, Opinion, Interview, Law) |
|---|---|---|---|---|
| El Comercio | 31 December 2002 | Spanish | Indicadores económicos, PERU ECONOMICO, APOYO COMUNICACIONES. | Informative |
| Gestión Online | 17 June 2019 | Spanish | MEF: “Empresas de bebidas bajaron el nivel de azúcar para pagar menos ISC ” | Informative |
| LatinFinance | 1 September 1994 | English | Fernando Bellido de Lama Carlos Saco-Vertiz Tudela | Informative |
| Reuters - Noticias Latinoamericanas | 1 September 1995 | Spanish | CIERRES MIXTOS EN BOLSA PERUANA. | Informative |
| Reuters - Noticias Latinoamericanas | 17 December 1997 | Spanish | ENTREVISTA-Lindley Perú busca socio para conquistar extranjero. | Informative |
| Perú Económico | 1 April 1998 | Spanish | LA RECAUDACION FISCAL, PERU ECONOMICO, APOYO COMUNICACIONES. | Informative |
| Semana Economica | 16 August 1998 | Spanish | ¿Menor demanda, mayor utilidad?, SEMANA ECONOMICA, APOYO COMUNICACIONES. | Informative |
| Semana Economica | 4 October 1998 | Spanish | Crecimiento. | Informative |
| Semana Economica | 17 January 1999 | Spanish | consumo masivo. | Informative |
| Semana Economica | 24 January 1999 | Spanish | Mensajes mixtos, SEMANA ECONOMICA, APOYO COMUNICACIONES. | Informative |
| Semana Economica | 11 April 1999 | Spanish | tributacion, SEMANA ECONOMICA, APOYO COMUNICACIONES. | Informative |
| Semana Economica | 26 April 1999 | Spanish | TU NO, SEMANA ECONOMICA, APOYO COMUNICACIONES. | Informative |
| Semana Economica | 26 April 1999 | Spanish | ¿TOCAMOS FONDO?, SEMANA ECONOMICA, APOYO COMUNICACIONES. | Informative |
| Semana Economica | 6 June 1999 | Spanish | "Este año se definirán los jugadores que quedarán en el mercado". | Interview |
| Semana Economica | 13 June 1999 | Spanish | "No hay peor error que caer en la inacción", SEMANA ECONOMICA, APOYO COMUNICACIONES. | Interview |
| Semana Economica | 2 August 1999 | Spanish | EPISODIO I - LA AMENAZA FANTASMA, SEMANA ECONOMICA, APOYO COMUNICACIONES. | Informative |
| Semana Economica | 21 September 1999 | Spanish | Sobrecostos, SEMANA ECONOMICA, APOYO COMUNICACIONES. | Informative |



Zuleta M, *et al. BMJ Glob Health* 2023; 8:e012024. doi: 10.1136/bmjgh-2023-012024

| Media Source | Date | Language | Title | Type of news (Informative, Opinion, Interview, Law) |
|---|---|---|---|---|
| Dow Jones International News | 27 September 1999 | English | Peru Bumps Up Gasoline, Cigarette Taxes | Informative |
| Semana Economica | 6 October 1999 | Spanish | Situación fiscal. | Informative |
| Semana Economica | 14 October 1999 | Spanish | ¿Fumas?, SEMANA ECONOMICA, APOYO COMUNICACIONES. | Informative |
| Perú Económico | 26 October 1999 | Spanish | ¿HABRA AJUSTE?, PERU ECONOMICO, APOYO COMUNICACIONES. | Informative |
| Perú Económico | 26 October 1999 | Spanish | Sustento, PERU ECONOMICO, APOYO COMUNICACIONES. | Opinion |
| Perú Económico | 1 January 2000 | Spanish | Retomar el paso, PERU ECONOMICO, APOYO COMUNICACIONES. | Opinion |
| Reuters - Noticias Latinoamericanas | 22 February 2000 | Spanish | Altos impuestos quitan aire a Backus, la mayor cervecera de Perú. | Informative |
| Semana Economica | 23 October 2000 | Spanish | Vicioso, SEMANA ECONOMICA, APOYO COMUNICACIONES. | Informative |
| Semana Economica | 29 October 2000 | Spanish | Dolores de cabeza, SEMANA ECONOMICA, APOYO COMUNICACIONES. | Informative |
| Perú Económico | 1 November 2000 | Spanish | Indicadores económicos, PERU ECONOMICO, APOYO COMUNICACIONES. | Informative |
| Reuters - Noticias Latinoamericanas | 1 November 2000 | Spanish | Backus de Perú reclama por elevado impuesto al consumo cerveza . | Informative |
| El País | 5 November 2000 | Spanish | La división en la Fuerzas Armadas frustró el compló contra el presidente Fujimori. Por FRANCESC RELEA, ENVIADO ESPECIAL. | Informative |
| Semana Economica | 14 January 2001 | Spanish | ¿Chau exoneraciones?, SEMANA ECONOMICA, APOYO COMUNICACIONES. | Informative |
| Reuters - Noticias Latinoamericanas | 15 January 2001 | Spanish | Titulares de la prensa peruana. | Informative |
| Semana Economica | 22 January 2001 | Spanish | A falta de beeper. | Opinion |
| Dow Jones International News | 22 January 2001 | English | Peru Repeals Heated Excise Tax On Beer ,Restores Flat Rate | Informative |
| Gestion | 24 January 2001 | English | Polar to increase stake in Backus & Johnston. | Informative |
| Semana Economica | 22 April 2001 | Spanish | ¿Sólo cerveceros?, SEMANA ECONOMICA, APOYO COMUNICACIONES. | Informative |
| Gestion | 24 May 2001 | English | Backus & Johnston to close down other plants if taxes are not reduced. | Informative |
| Perú Económico | 1 September 2001 | Spanish | Presiones fiscales, PERU ECONOMICO, APOYO COMUNICACIONES. | Informative |

| Media Source | Date | Language | Title | Type of news (Informative, Opinion, Interview, Law) |
|---|---|---|---|---|
| Semana Economica | 10 September 2001 | Spanish | Non mi piace, SEMANA ECONOMICA, APOYO COMUNICACIONES. | Informative |
| Reuters - Noticias Latinoamericanas | 27 September 2001 | Spanish | Titulares de la prensa peruana. | Informative |
| Semana Economica | 30 September 2001 | Spanish | Orden en la casa, SEMANA ECONOMICA, APOYO COMUNICACIONES. | Informative |
| Semana Economica | 18 November 2001 | Spanish | Discusiones íntimas, SEMANA ECONOMICA, APOYO COMUNICACIONES. | Opinion |
| Semana Economica | 18 November 2001 | Spanish | Polémica chela, SEMANA ECONOMICA, APOYO COMUNICACIONES. | Informative |
| Gestion | 21 November 2001 | English | Backus to close down plants if sales decrease. | Informative |
| Semana Economica | 7 December 2001 | Spanish | Efectos, SEMANA ECONOMICA, APOYO COMUNICACIONES. | Informative |
| Semana Economica | 7 December 2001 | Spanish | perfil, SEMANA ECONOMICA, APOYO COMUNICACIONES. | Informative |
| Semana Economica | 11 February 2002 | Spanish | ¡Buen negocio!, SEMANA ECONOMICA, APOYO COMUNICACIONES. | Informative |
| Reuters - Noticias Latinoamericanas | 4 April 2002 | Spanish | Recaudación mayor ente tributario Perú cae 12,9 pct real/marzo. | Informative |
| Gestion | 4 June 2002 | English | Consumption of liquor drops by 20%. | Informative |
| Reuters - Noticias Latinoamericanas | 4 July 2002 | Spanish | Ventas de cervecera Backus (BKJi.LM) sube 14 pct en 1er semestre. | Informative |
| Gestion | 9 July 2002 | English | Beer profits up more than 30%. | Informative |
| Gestion | 22 July 2002 | English | Backus profits up 99% in first six months. | Informative |
| Semana Económica | 23 August 2002 | Spanish | Más que una cerveza , SEMANA ECONOMICA, APOYO COMUNICACIONES. | Informative |
| Agencia Mexicana de Noticias, NOTIMEX | 30 August 2002 | Spanish | AUMENTAN IMPUESTOS A LOS COMBUSTIBLES Y CERVEZAS EN PERU. | Informative |
| Reuters - Noticias Latinoamericanas | 31 August 2002 | Spanish | Perú incrementa ingresos 250 mlns soles con alza de impuestos. | Informative |
| Dow Jones News Service | 2 September 2002 | English | Peru's August Tax, Customs Haul Rises 7.9% - Sunat | Informative |

| Media Source | Date | Language | Title | Type of news (Informative, Opinion, Interview, Law) |
|---|---|---|---|---|
| Reuters - Noticias Latinoamericanas | 3 September 2002 | Spanish | Peruana Backus teme baja de ventas por aumento impuesto cerveza . | Informative |
| Reuters - Noticias Latinoamericanas | 6 September 2002 | Spanish | Perú lanzará en octubre emisión de 500 mln dlrs en bonos. | Informative |
| Reuters - Noticias Latinoamericanas | 6 September 2002 | Spanish | Perú planea bonos externos por 500 mlls dlrs a fines de octubre. | Informative |
| Semana Económica | 8 September 2002 | Spanish | oido en la calle, SEMANA ECONOMICA, APOYO COMUNICACIONES. | Informative |
| Reuters - Noticias Latinoamericanas | 9 September 2002 | Spanish | Bolsa peruana cierra mixta, cae cervecera Backus y Johnston. | Informative |
| Gestion | 9 September 2002 | English | Tobacco tax increase intended to create jobs. | Informative |
| Reuters - Noticias Latinoamericanas | 10 September 2002 | Spanish | Bolsa Perú cierra al alza por avance de mineras, atento a EE.UU. | Informative |
| Semana Económica | 16 September 2002 | Spanish | oido en la calle, SEMANA ECONOMICA, APOYO COMUNICACIONES. | Informative |
| Gestion | 19 September 2002 | English | Cisneros proposes Backus exports to Venezuela. | Informative |
| Gestion | 25 November 2002 | English | Wine sales up this year. | Informative |
| El Comercio | 27 November 2002 | Spanish | Gravarían las pensiones mayores de 5 mil soles mensuales. | Informative |
| El Comercio | 2 January 2003 | Spanish | Sector de electrodomésticos pierde US$50 millones por contrabando. | Informative |
| El Comercio | 3 January 2003 | Spanish | La recaudación tributaria creció 23,9% en diciembre. | Informative |
| Gestion | 10 January 2003 | English | Backus expects continued sales increase in 2003. | Informative |
| El Comercio | 18 January 2003 | Spanish | Recaudación. | Informative |
| El Comercio | 21 January 2003 | Spanish | Cigarrillos pagarán un impuesto al consumo de 30%. | Informative |
| Economist Intelligence Unit - ViewsWire | 24 January 2003 | | Peru economy: Manufacturing update | Informative |
| El Comercio | 4 March 2003 | Spanish | La recaudación aumentó un 26,4% en febrero. | Informative |
| Gestion | 25 March 2003 | English | Backus increase storage capacity. | Informative |
| El Comercio | 2 April 2003 | Spanish | El Impuesto Vehicular crece 16,1% en marzo. | Informative |

| Media Source | Date | Language | Title | Type of news (Informative, Opinion, Interview, Law) |
|---|---|---|---|---|
| Semana Económica | 14 April 2003 | Spanish | perfil, SEMANA ECONOMICA, APOYO COMUNICACIONES. | Informative |
| El Comercio | 3 June 2003 | Spanish | Crece la recaudación tributaria un 21,8% respecto a mayo de 2002. | Informative |
| Agencia EFE - Servicio Económico | 16 June 2003 | Spanish | Gobierno peruano reorienta 200 millones de dólares de presupuesto. | Informative |
| Reuters - Noticias Latinoamericanas | 16 June 2003 | Spanish | Perú eleva impuestos y baja sueldos para ajustar presupuesto. | Informative |
| Reuters - Noticias Latinoamericanas | 16 June 2003 | Spanish | ACTUALIZA 1-Perú ajustará presupuesto con más impuestos. | Informative |
| Dow Jones International News | 16 June 2003 | English | Peru's Fin Minister Unveils Tax, Spending Overhaul | Informative |
| Agencia Mexicana de Noticias, NOTIMEX | 17 June 2003 | Spanish | CONSIDERAN QUE MEDIDAS TRIBUTARIAS AUMENTARAN CONTRABANDO EN PERU. | Informative |
| Agencia Mexicana de Noticias, NOTIMEX | 17 June 2003 | Spanish | (Previsión) - GENERA AMPLIO RECHAZO POPULAR "PAQUETAZO" TRIBUTARIO EN PERU. | Informative |
| Agence France Presse | 17 June 2003 | Spanish | Ola de rechazo a medidas tributarias planteadas por gobierno de Toledo. | Informative |
| Agence France Presse | 17 June 2003 | Spanish | Las 10 controvertidas medidas tributarias en Perú. | Informative |
| El Comercio | 17 June 2003 | Spanish | Gobierno lanza aumento de impuestos para aumentar la recaudación. | Informative |
| Reuters - Noticias Latinoamericanas | 19 June 2003 | Spanish | Bolsa peruana cae al cierre, sigue desánimo por plan tributario. | Informative |
| Reuters News | 19 June 2003 | English | BAT puts Peru spending on hold after tax increase. | Informative |
| Reuters - Noticias Latinoamericanas | 20 June 2003 | Spanish | Backus rebaja precio de cerveza en Perú, pese alza de impuesto. | Informative |
| Reuters - Noticias Latinoamericanas | 20 June 2003 | Spanish | Bolsa Perú cierra al alza con apoyo títulos bancarios y Backus. | Informative |
| El Comercio | 20 June 2003 | Spanish | Ministro pide al Congreso alternativas a reformas tributarias rechazadas. | Informative |
| El Comercio | 20 June 2003 | Spanish | Backus sugiere reducir del precio de la cerveza de venta al público. | Informative |
| Portafolio | 23 June 2003 | Spanish | Backus & Johnston reduce precios para incentivar consumo de cerveza . | Informative |
| Reuters - Noticias Latinoamericanas | 24 June 2003 | Spanish | Bolsa peruana cierra a la baja en jornada con volumen pobre. | Informative |

| Media Source | Date | Language | Title | Type of news (Informative, Opinion, Interview, Law) |
|---|---|---|---|---|
| El Comercio | 1 July 2003 | Spanish | Lucha contra el contrabando generaría US$ 300 millones al Estado. | Informative |
| Agencia Mexicana de Noticias, NOTIMEX | 2 July 2003 | Spanish | FRACASA GOBIERNO PERUANO EN INTENTO DE APLICAR IMPUESTOS A TELEFONIA. | Informative |
| Agence France Presse | 2 July 2003 | Spanish | British American Tobacco prevé despidos ante incremento de impuestos en Perú. | Informative |
| El Comercio | 9 July 2003 | Spanish | British American Tobacco acapara el 95% del mercado local de cigarrillos . | Interview |
| El Comercio | 14 July 2003 | Spanish | Opinión - Impuestos se enfrentan a manejos insuficientes. | Opinion |
| Reuters - Noticias Latinoamericanas | 1 August 2003 | Spanish | Bolsa peruana cierra al alza por avance de mineras. | Informative |
| El Comercio | 6 August 2003 | Spanish | Gobierno baja impuesto al consumo para cigarrillosy cerveza . | Informative |
| Diario Financiero Online | 6 August 2003 | Spanish | Gobierno peruano dictaminó rebaja selectiva de impuestos al cigarrillo . | Informative |
| Reuters - Noticias Latinoamericanas | 6 August 2003 | Spanish | Titulares de la prensa peruana. | Informative |
| Gestion | 26 August 2003 | English | British American Tobacco may leave Peru if tax situation does not improve. | Informative |
| El Comercio | 4 November 2003 | Spanish | Recaudación creció 6,6% en octubre. | Informative |
| El Comercio | 5 November 2003 | Spanish | Esperan que nuevas disposiciones tributarias estén listas para diciembre. | Informative |
| Dow Jones International News | 5 November 2003 | English | Peru's Fin Min Confirms Bank Transaction Tax To Proceed | Informative |
| El Comercio | 11 November 2003 | Spanish | Gobierno reduciría impuesto al comsumo para cigarrillos . | Informative |
| El Comercio | 12 December 2003 | Spanish | Sector empresarial esperan la derogación de impuesto a transacciones. | Informative |
| El Comercio | 19 December 2003 | Spanish | Modificaciones legislativas analizan tasas de tributos. | Informative |
| Latin America News Digest | 9 February 2004 | English | Peruvian Govt Reduces Cigarette Tax to 30 Pct | Informative |
| El Comercio | 9 February 2004 | Spanish | Gobierno reduce impuesto a cigarrillos de 37,5 a 30%. | Informative |

| Media Source | Date | Language | Title | Type of news (Informative, Opinion, Interview, Law) |
|---|---|---|---|---|
| El Comercio | 1 March 2004 | Spanish | Vitivinícolas piden modificar la forma de cálculo de impuesto al consumo | Informative |
| Dow Jones Emerging Markets Report | 10 March 2004 | English | Peru's Feb Tax Haul Rises 10.1% Over Yr; Bank Tax Starts | Informative |
| Latin America News Digest | 17 March 2004 | English | British American Tobacco Renews Investments in Peru | Informative |
| El Comercio | 17 March 2004 | Spanish | British American Tobacco retoma inversiones en el país | Informative |
| Latin America News Digest | 5 April 2004 | English | Peruvian Tax Collections up 13.1 Pct Y/Y Mar 2004 | Informative |
| El Comercio | 28 May 2004 | Spanish | Ministro de Economía considera díficil luchar contra contrabando de cerveza | Informative |
| Business News Americas | 1 June 2004 | Spanish | Quijandría define plan de estabilidad para precios de combustible | Informative |
| El Comercio | 10 June 2004 | Spanish | Sector industrial pide bajar el margen de rentabilidad al contrabando | Informative |
| NoticiasFinancieras | 29 July 2004 | Spanish | Backus reduce 61,2% utilidades netas sin consolidar en primer semestre | Informative |
| El Comercio | 30 July 2004 | Spanish | Impuesto Selectivo al Consumo del pisco es de 1,50 soles por litro en Perú | Informative |
| El Comercio | 8 December 2004 | Spanish | Ministerio de Economía reduce al 12% arancel a cigarrillos | Informative |
| El Comercio | 3 March 2005 | Spanish | Recaudación tributaria subió 6,9% en febrero | Informative |
| Portafolio | 18 May 2005 | Spanish | Empresarios colombianos intensifican negocios en Ecuador, Perú y Panamá | Informative |
| El Comercio | 7 December 2005 | Spanish | Recaudación de tributos internos sube 16,2% durante noviembre | Informative |
| El Comercio | 3 March 2006 | Spanish | Sistema tributario del país es bueno pero puede mejorar según FMI | Informative |
| Reuters - Noticias Latinoamericanas | 30 March 2006 | Spanish | ACTUALIZA 2-SUMMIT-SABMiller invertirá 90 mln dlr en Backus Perú | Informative |
| Reuters - Noticias Latinoamericanas | 3 May 2006 | Spanish | Cervecería Backus y Johnston Perú reduce 6 pct utilidad 1er trim | Informative |
| El Comercio | 10 May 2006 | Spanish | Otro campo | Informative |
| El Comercio | 8 June 2006 | Spanish | Recaudación interna aumenta 29% en mayo | Informative |



Zuleta M, *et al. BMJ Glob Health* 2023; 8:e012024. doi: 10.1136/bmjgh-2023-012024

| **Media Source** | **Date** | **Language** | **Title** | **Type of news (Informative, Opinion, Interview, Law)** |
|---|---|---|---|---|
| El Comercio | 24 June 2006 | Spanish | Xxxxxx xxxxxxxxxxxx xxx xxxxxxx xx xxxxxxxxxxxx xxx xxxxxxx xxxxx x | Informative |
| Reuters - Noticias Latinoamericanas | 31 July 2006 | Spanish | Ganancias peruana Backus crecen un 52 pct en segundo trimestre | Informative |
| El Comercio | 29 September 2006 | Spanish | Xxxxxx xxxxxxxx xxxxx xxxxxxxxx xxxxxxxx xxxx | Informative |
| El Comercio | 6 October 2006 | Spanish | El Estado espera recaudar este gaño | Informative |
| Business Peru | 13 October 2006 | Spanish | Mercado del pisco peruano crece a un ritmo anual del 20% | Informative |
| Reuters - Noticias Latinoamericanas | 30 October 2006 | Spanish | ACTUALIZA 1-Ganancia peruana Backus crece 49,8 pct en 3er trim | Informative |
| Business Peru | 29 December 2006 | Spanish | Espirituoso repunte | Informative |
| Business Peru | 11 January 2007 | Spanish | Un 60% del licor que consume en el país es informal, dice informe | Informative |
| Reuters - Noticias Latinoamericanas | 14 February 2007 | Spanish | ACTUALIZA 1-Ganancia de Backus Perú crece 84 pct en 4to trim | Informative |
| El Comercio | 4 July 2007 | Spanish | Opinión-¿Cómo impulsar el uso de biocombustibles? | Opinion |
| El Comercio | 17 April 2008 | Spanish | Recaudación tributaria en marzo crece 18,4% | Informative |
| Semana Económica | 8 March 2009 | Spanish | Opiniones selectivas | Informative |
| El Comercio | 17 July 2009 | Spanish | Apoyo propone cambios al impuesto aplicado a los licores | Informative |
| Reuters - Noticias Latinoamericanas | 30 July 2009 | Spanish | Utilidad de cervecería Backus crece 26,7 pct en 2do trim | Informative |
| Semana Económica | 17 January 2010 | Spanish | LA SEMANA ECONÓMICA… QUE PASÓ | Informative |
| El Comercio | 23 January 2010 | Spanish | Subirán en 50% ISC a los cigarrillos | Informative |
| Semana Económica | 24 January 2010 | Spanish | LA SEMANA ECONÓMICA… QUE PASÓ | Informative |
| Agence France Presse | 31 January 2010 | Spanish | Perú gasta unos USD 103 millones en tratamiento de cáncer asociado al tabaco | Informative |
| El Comercio | 4 February 2010 | Spanish | Maximizando el daño | Opinion |
| El Comercio | 9 February 2010 | Spanish | Con su humo a otra parte | Opinion |
| El Comercio | 31 March 2010 | Spanish | Tabacalera estima que venderá menos | Informative |

| Media Source | Date | Language | Title | Type of news (Informative, Opinion, Interview, Law) |
|---|---|---|---|---|
| El Comercio | 20 April 2010 | Spanish | Mayor captación de tributos durante marzo | Informative |
| El Comercio | 11 May 2010 | Spanish | Modificarían ISC a bebidas alcohólicas | Informative |
| NoticiasFinancieras | 21 May 2010 | Spanish | Nuevo impuesto puede afectar competencia en el mercados de cervezas | Informative |
| Semana Económica | 23 May 2010 | Spanish | LA SEMANA ECONÓMICA... QUE PASÓ | Informative |
| El Comercio | 1 June 2010 | Spanish | Las cifras | Informative |
| El Comercio | 8 June 2010 | Spanish | Importación de cigarrillos cae 18% por aumento del ISC | Informative |
| Agencia Andina | 3 February 2011 | English | Peru: Beer market expands 8% to US$1.2bil in 2011 | Informative |
| El Comercio | 16 May 2013 | Spanish | Nuevo impuesto a licores se trasladará al consumidor | Informative |
| Semana Económica | 19 May 2013 | Spanish | LA SEMANA ECONÓMICA... QUE PASÓ | Informative |
| El Comercio | 23 October 2013 | Spanish | Ambev Perú solicita el cese colectivo de 250 empleados | Informative |
| El Comercio | 23 November 2013 | Spanish | Menor crecimiento en bebidas | Informative |
| Gestión Online | 27 February 2014 | Spanish | Aumento del ISC terminó favoreciendo a nuestra competencia, sostiene Ambev | Informative |
| El Comercio | 28 February 2014 | Spanish | Ambev Perú apunta a duplicar su parte del mercado local en tres años | Informative |
| Valor Futuro | 22 May 2014 | Spanish | Importaciones peruanas de bebidas alcohólicas crecerán 10,4% el 2014; vinos +6,5% - Maximixe | Informative |
| Gestión Online | 22 May 2014 | Spanish | Importación de bebidas alcohólicas crecería 10.4% este año, estima Maximixe | Informative |
| Gestión Online | 2 June 2014 | Spanish | Macro Región Oriente aumentó su recaudación de tributos internos en 2.6% | Informative |
| El Comercio | 4 June 2014 | Spanish | Ambev le hará frente a Backus con un portafolio más amplio | Informative |
| El Comercio | 24 June 2014 | Spanish | Venta de licor informal crece 4 puntos porcentuales en el 2013 | Informative |
| Esmerk Latin American News | 24 June 2014 | English | Peru: Alcoholic beverage companies call for modifications to ISC tax | Informative |
| Gestión | 1 July 2014 | Spanish | "Licores informales crecieron 7 puntos por aumento de ISC " | Informative |
| Esmerk Latin American News | 1 July 2014 | English | Peru: Informal market in liquor sector grows under new tax system | Informative |

| **Media Source** | **Date** | **Language** | **Title** | **Type of news (Informative, Opinion, Interview, Law)** |
|---|---|---|---|---|
| Perú 21 | 20 July 2014 | Spanish | “Medidas son del Tercer Mundo” | Informative |
| El Comercio | 21 July 2014 | Spanish | Anpay tiene a Lima en la mira | Informative |
| Esmerk Latin American News | 21 July 2014 | English | Peru: Grupo Torvisco repositions Anpay beer brand in Lima | Informative |
| El Comercio | 3 August 2014 | Spanish | ¿Dónde está la ?Chela? más Barata? | Informative |
| El Comercio | 8 August 2014 | Spanish | Tributación del sector industrial cayó 8,8% en el primer semestre | Informative |
| El Comercio | 11 August 2014 | Spanish | Economy | Informative |
| Gestión Online | 13 August 2014 | Spanish | Producción peruana de vinos crecería 5.4% este año, estima Maximixe | Informative |
| Gestión Online | 28 August 2014 | Spanish | La reforma equivocada del impuesto a las cervezas | Opinion |
| Gestión | 19 September 2014 | Spanish | Hay consenso para uniformizar ISC que se aplica a la cerveza | Informative |
| Perú 21 Online | 21 September 2014 | Spanish | Comex: Impuesto a la cerveza en Perú es uno de los más altos del mundo | Informative |
| Perú 21 Online | 23 September 2014 | Spanish | Congreso busca realizar cambios en el impuesto a la cerveza | Informative |
| Gestión Online | 2 October 2014 | Spanish | MEF defiende sistema del ISC que grava a cervezas | Informative |
| Gestión Online | 2 October 2014 | Spanish | La cerveza y el impuesto que evita la competencia por precios | Informative |
| Gestión Online | 3 October 2014 | Spanish | SNI: Altos impuestos a los licores fomentan informalidad | Informative |
| Gestión | 6 October 2014 | Spanish | Congreso dividido por proyecto que elimina ISC mixto a las cervezas | Informative |
| Esmerk Latin American News | 8 October 2014 | English | Peru: ISC tax increase only benefited informal alcohol market | Informative |
| El Comercio | 9 October 2014 | Spanish | Backus afirma que los cambios en el ISC también le afectaron | Informative |
| Gestión | 9 October 2014 | Spanish | Confianza y predictibilidad: Pilares de un sistema tributario maduro | Informative |
| Esmerk Latin American News | 9 October 2014 | English | Peru: Tax changes also affected SABMiller subsidiary | Informative |
| Gestión Online | 10 October 2014 | Spanish | Modificación del ISC a las cervezas se verá a fin de mes | Informative |

| Media Source | Date | Language | Title | Type of news (Informative, Opinion, Interview, Law) |
|---|---|---|---|---|
| Esmerk Latin American News | 10 October 2014 | English | Peru: Congress to discuss changes to ISC tax on beer | Informative |
| Gestion - SABI (Abstracts) | 11 October 2014 | English | Peru: Beer tax clears bars | Informative |
| Perú 21 | 15 October 2014 | Spanish | Regulación económica y tirole | Informative |
| Esmerk Latin American News | 21 October 2014 | English | Peru: Informal sales of alcohol on the rise | Informative |
| Gestión | 22 October 2014 | Spanish | Informalidad y políticas públicas | Opinion |
| El Comercio | 23 October 2014 | Spanish | Productores artesanales en contra de cambio en el ISC a la cerveza | Informative |
| Gestión Online | 23 October 2014 | Spanish | SNI: Precio de licores subió e informalidad se incrementó por cambios en el ISC | Informative |
| Esmerk Latin American News | 23 October 2014 | English | Peru: Microbrewery opposes taxation changes | Informative |
| El Comercio | 31 October 2014 | Spanish | Backus muestra que Ambev mejoró su cuota de mercado con el actual ISC | Informative |
| Gestión Online | 17 November 2014 | Spanish | “Nuestra fortaleza radica hoy en un portafolio diferenciado” | Interview |
| Perú 21 Online | 26 November 2014 | Spanish | "Desde Palacio me vetan en el caso Belaunde" | Interview |
| Gestión Online | 3 December 2014 | Spanish | Cerveceros artesanales se unen para fortalecer sus condiciones de competencia | Informative |
| El Comercio | 16 December 2014 | Spanish | La SNI busca acercamiento con el MEF para bajar impuesto a licores | Informative |
| Esmerk Latin American News | 16 December 2014 | English | Peru: SNI seeks talks with MEF to lower taxes on liquors | Informative |
| Gestión Online | 9 February 2015 | Spanish | Perucámaras: Recaudación tributaria en Macro Región Oriente creció 6.2% en el 2014 | Informative |
| ValorFuturo | 23 February 2015 | Spanish | Recaudación tributaria en Macro Región Sur de Perú aumentó en 14,6% el 2014/13 -PerúCámaras | Informative |
| Gestión Online | 23 February 2015 | Spanish | Recaudación tributaria en Macro Región Sur aumentó en 14,6% en el 2014 | Informative |

| Media Source | Date | Language | Title | Type of news (Informative, Opinion, Interview, Law) |
|---|---|---|---|---|
| Gestión Online | 26 February 2015 | Spanish | LC Group trae Guaraná Antarctica al Perú y prevé vender más de 700,000 unidades este año | Informative |
| Gestión Online | 3 March 2015 | Spanish | Comisión de Economía debatirá garantías mobiliarias | Informative |
| Gestión Online | 11 March 2015 | Spanish | Ministerio de Salud propondrá subir el tributo a los cigarrillos | Informative |
| Gestión Online | 14 March 2015 | Spanish | Resumen económico de la semana: BCR adelanta dato del PBI y Petroperú ya no explotaría lotes | Informative |
| Gestión | 16 March 2015 | Spanish | Impresa | Informative |
| El Comercio | 4 April 2015 | Spanish | pérdidas y costos del tabaquismo | Informative |
| Gestión | 20 April 2015 | Spanish | Sunat: Ingresos por IGV interno se recuperaron en marzo y crecieron 1.9% | Informative |
| Gestión Online | 6 June 2015 | Spanish | Hoy es el Día del Ron Peruano, ¿Sabe cuánto es su consumo per cápita? | Informative |
| Semana Económica | 6 September 2015 | Spanish | Señales de humo | Informative |
| Gestión Online | 11 September 2015 | Spanish | Recaudación de IGV Interno crece 4.4% en agosto impulsado por empresas manufactureras | Informative |
| Gestión Online | 17 September 2015 | Spanish | Cerveceros artesanales peruanos proyectan vender 1 millón de litros en 2015 | Informative |
| Gestión | 12 October 2015 | Spanish | Fujimorismo busca en Congreso bajar precio de la cerveza | Informative |
| Esmerk Latin American News | 12 October 2015 | English | Peru: Fuerza Popular introduces bill to lower the price of beer | Informative |
| Gestión Online | 15 October 2015 | Spanish | Venta de SABMiller abre espacio para aumento de precios y nuevas marcas | Informative |
| El Comercio | 3 November 2015 | Spanish | Consumo de alcohol ilegal alcanza los US$584 mlls. | Informative |
| Gestión | 3 November 2015 | Spanish | Cambios tributarios en el ISC habrían impulsado el mercado ilegal de alcohol | Informative |
| Gestión Online | 17 November 2015 | Spanish | Hoy aprobarían cambios en el ISC que se aplica a la cerveza | Informative |

| Media Source | Date | Language | Title | Type of news (Informative, Opinion, Interview, Law) |
|---|---|---|---|---|
| Esmerk Latin American News | 17 November 2015 | English | Peru: ISC changes for beer debated at the Consumer Defence Commission | Informative |
| Gestión Online | 17 November 2015 | Spanish | Comisión de Defensa al Consumidor aprobaría cambios en ISC a la cerveza | Informative |
| Gestión | 18 November 2015 | Spanish | PERISCOPIO ECONÓMICO | Informative |
| Gestión Online | 28 November 2015 | Spanish | Grupo AJE dispuesto a competir en mercado de cerveza si se modifica sistema de impuestos | Informative |
| Gestión Online | 30 November 2015 | Spanish | Aje Group a favor de cambio de ISC a la cerveza | Informative |
| Gestion - SABI (Abstracts) | 30 November 2015 | English | Peru: Aje would only consider beer investments under a new taxation system | Informative |
| Esmerk Latin American News | 30 November 2015 | English | Peru: Aje Group in favour of changes to ISC tax on beer | Informative |
| Gestión | 1 December 2015 | Spanish | PERISCOPIO ECONÓMICO | Informative |
| El Comercio | 2 December 2015 | Spanish | El posible cambio del ISC desata una intensa guerra entre cerveceras | Informative |
| Gestión Online | 2 December 2015 | Spanish | Piden eliminar sistema mixto del ISC que se aplica a los vinos | Informative |
| Esmerk Latin American News | 2 December 2015 | English | Peru: Beer tax change debate at CDC is postponed again | Informative |
| Gestión Online | 3 December 2015 | Spanish | ComexPerú reclama ISC único para todas las bebidas alcohólicas | Informative |
| Gestión Online | 6 December 2015 | Spanish | Comex: Régimen del ISC a cervezas se volvería discriminatorio si se aprueba propuesta del Congreso | Informative |
| Gestión Online | 11 April 2016 | Spanish | Recaudación tributaria en Macro Región Oriente aumentó más de 7% | Informative |
| Esmerk Latin American News | 20 April 2016 | English | Peru: CDC approves modifications to ISC tax in beer sector | Informative |
| Gestión Online | 20 April 2016 | Spanish | Jubilados de AFP podrán retirar sus fondos dentro de pocos días y BCR plantea seguir con reforma del SPP | Informative |
| El Comercio | 21 April 2016 | Spanish | MEF afirma que el pleno aprobaría leyes sin sustento técnico | Informative |

| Media Source | Date | Language | Title | Type of news (Informative, Opinion, Interview, Law) |
|---|---|---|---|---|
| Esmerk Latin American News | 21 April 2016 | English | Peru: State to lose PEN 200mn a year in alcohol tax | Informative |
| Esmerk Latin American News | 22 April 2016 | English | Peru: Beer tax bill to encourage price competition | Informative |
| El Comercio | 23 April 2016 | Spanish | Leyes con burbujas pero sin puntería | Informative |
| Gestión Online | 24 April 2016 | Spanish | Comex: proyecto del Congreso para cambiar el ISC a la cerveza es “irresponsable” | Informative |
| Gestión Online | 25 April 2016 | Spanish | Eliminación de impuesto mixto a la cerveza no elevaría la producción en un mercado concentrado | Informative |
| Gestión Online | 25 April 2016 | Spanish | Perucámaras: Recaudación tributaria en Macro Región Sur cayó 1% | Informative |
| Gestión Online | 2 May 2016 | Spanish | Reducir el ISC a la cerveza artesanal evitará que desaparezca esta bebida, dicen sus fabricantes | Informative |
| Gestión Online | 5 May 2016 | Spanish | ISC a cigarrillos se eleva en 157% y EsSalud suspende nuevas afiliaciones de trabajadores independientes | Informative |
| El Mercurio | 6 May 2016 | Spanish | Perú eleva impuestos a combustibles y cigarrillos | Informative |
| Perú 21 Online | 6 May 2016 | Spanish | Ministerio de Salud: 'Incremento del impuesto al tabaco reduciría en 8% el consumo de cigarrillos ' | Informative |
| Agence France Presse | 7 May 2016 | Spanish | Perú sube impuestos al tabaco y combustibles en base a índice de nocividad | Informative |
| Gestión Online | 8 May 2016 | Spanish | Australianos pagarán US$ 30 por una cajetilla de cigarros | Informative |
| El Comercio | 9 May 2016 | Spanish | El futuro del control del tabaco en el Perú | Informative |
| People’s Daily Online | 10 May 2016 | Spanish | Organización Panamericana de la Salud destaca medida antitabaco para jóvenes en Perú | Informative |
| Gestión Online | 11 May 2016 | Spanish | El aumento ISC de los cigarrillos | Informative |
| Gestión Online | 11 May 2016 | Spanish | Recaudación IGV reporta crecimiento real de 2.2% en abril | Informative |
| El Comercio | 13 May 2016 | Spanish | El ISC y la reducción de daños | Opinion |
| Gestión Online | 14 May 2016 | Spanish | Comex: alza del ISC a los cigarros requiere medidas complementarias para reducir su consumo | Informative |
| Semana Económica | 15 May 2016 | Spanish | El falso dilema del impuesto selectivo | Opinion |



Zuleta M, *et al. BMJ Glob Health* 2023; 8:e012024. doi: 10.1136/bmjgh-2023-012024

| Media Source | Date | Language | Title | Type of news (Informative, Opinion, Interview, Law) |
|---|---|---|---|---|
| Gestión Online | 16 May 2016 | Spanish | Perucámaras: Recaudación tributaria en Macro Región Centro cayó 5.3% | Informative |
| Perú 21 Online | 29 May 2016 | Spanish | El 50% de jóvenes que prueba el cigarro se engancha con su uso | Informative |
| Reuters - Noticias Latinoamericanas | 1 June 2016 | Spanish | Perú anota inflación de 0,21 pct en mayo, tasa anualizada se desacelera a un 3,54 pct: Gobierno | Informative |
| El Comercio | 1 June 2016 | Spanish | Externalidades negativas | Opinion |
| Gestión Online | 2 June 2016 | Spanish | Editorial: A revisar los números | Informative |
| Gestión Online | 9 June 2016 | Spanish | Venta de licores se recupera de la 'resaca' y crecería 6% en el 2016, estima la CCL | Informative |
| Gestión Online | 9 June 2016 | Spanish | Recaudación por Impuesto a la Renta del Gobierno Central aumentó 11% en mayo | Informative |
| Perú 21 Online | 9 June 2016 | Spanish | Sector licores crecería 6% este 2016, según el Gremio de Vinos y Licores | Informative |
| ValorFuturo | 9 June 2016 | Spanish | Recaudación tributaria de Perú creció 1,7% en términos reales en May 2016/15;Imp. a la Renta +11% | Informative |
| Reuters - Noticias Latinoamericanas | 1 July 2016 | Spanish | Perú anota inflación de 0,14 pct en junio, tasa anualizada se desacelera a un 3,34 pct:Gobierno | Informative |
| Perú 21 Online | 1 July 2016 | Spanish | INEI: Inflación en junio fue de 0.14%, la segunda más baja del año | Informative |
| El Comercio | 5 July 2016 | Spanish | Leyes que se hacen humo | Opinion |
| Gestión Online | 12 July 2016 | Spanish | Sunat: Recaudación de IGV interno cae 5.1% impulsado por sector manufactura | Informative |
| ValorFuturo | 12 July 2016 | Spanish | Ingresos tributarios de Perí cayeron en términos reales en 1,1% en Jun 2016; sumaron PEN 8.046 MM | Informative |
| Gestión Online | 12 July 2016 | Spanish | Sunat: Recaudación de IGV interno cae 5.1% afectado por sector manufactura | Informative |
| Gestión Online | 7 September 2016 | Spanish | Sunat: Ingresos tributarios caen 2.7% en agosto por menor pago del Impuesto a la Renta | Informative |
| Gestión Online | 5 October 2016 | Spanish | Cerveceros artesanales del Perú venderán más de un millón de litros en 2016 | Informative |
| Gestión Online | 12 October 2016 | Spanish | Ingresos tributarios del Gobierno Central solo aumentaron en S/ 257 millones en setiembre | Informative |



Zuleta M, *et al. BMJ Glob Health* 2023; 8:e012024. doi: 10.1136/bmjgh-2023-012024

| Media Source | Date | Language | Title | Type of news (Informative, Opinion, Interview, Law) |
|---|---|---|---|---|
| Gestión Online | 10 November 2016 | Spanish | Recaudación del IGV suma S/ 4,592 millones y crece 2.6% en octubre | Informative |
| Gestión Online | 16 November 2016 | Spanish | Fuerza Popular propone diferentes tasas de ISC para la cerveza industrial y artesanal | Informative |
| El Comercio | 19 November 2016 | Spanish | CongresoPEDIA | Informative |
| El Comercio | 21 November 2016 | Spanish | Los fuji-tecnócratas | Opinion |
| El Comercio | 7 December 2016 | Spanish | Saavedra 2021 | Opinion |
| ValorFuturo | 12 December 2016 | Spanish | Recaudación tributaria de Perú cayó 3,5% en Nov 2016/15 por menor Impuesto a la Renta (-5,1%)-Sunat | Informative |
| Gestión Online | 12 January 2017 | Spanish | Editorial: Inflación cuasi controlada | Opinion |
| Gestión Online | 12 January 2017 | Spanish | Sunat: ingresos tributarios del gobierno central crecieron 0.1% el 2016 | Informative |
| El Comercio | 23 January 2017 | Spanish | Pernod Ricard traería whisky de US$1.000 | Informative |
| Peru Reports | 26 January 2017 | English | Peru’s craft brewers face high barriers to compete with beer monopoly | Informative |
| Gestión Online | 27 January 2017 | Spanish | Más de 420 millones de cigarrillos ingresaron al Perú de contrabando en el 2016 | Informative |
| Gestión Online | 7 March 2017 | Spanish | Editorial: ¿Quién paga impuestos? | Opinion |
| Gestión Online | 31 May 2017 | Spanish | Cuando el humo de los cigarrillos también daña a la economía | Informative |
| Esmerk Latin American News | 14 June 2017 | English | Peru: Beer consumption climbed in first five months of 2017 | Informative |
| El Comercio | 19 July 2017 | Spanish | Visiones presidenciales | Opinion |
| El Comercio | 20 September 2017 | Spanish | Lo que no cuesta, no obliga | Opinion |
| Gestión Online | 5 October 2017 | Spanish | Más de 30 cervecerías artesanales participarán en Lima Beer Week | Informative |
| Gestión Online | 15 December 2017 | Spanish | BAT innova sus modelos de negocio y distribución, y apuesta por el cigarrillo electrónico | Informative |

| Media Source | Date | Language | Title | Type of news (Informative, Opinion, Interview, Law) |
|---|---|---|---|---|
| El Comercio | 21 February 2018 | Spanish | Comisión Permanente debatirá proyectos de impacto económico | Informative |
| Esmerk Latin American News | 17 April 2018 | English | Peru: Congress to debate bill to reduce tax for small breweries | Informative |
| Gestión Online | 30 April 2018 | Spanish | Subirían tasas del ISC a tabaco , alcohol y combustibles, pero bajaría para otros | Informative |
| ValorFuturo | 30 April 2018 | Spanish | David Tuesta (MEF): Subirían tasas del ISC a tabaco , alcohol y combustibles en Perú | Informative |
| Gestión Online | 2 May 2018 | Spanish | ISC : ¿Cómo obtener S/ 1,342 millones adicionales para este año? | Informative |
| Gestión Online | 3 May 2018 | Spanish | Alza del ISC a combustibles, cigarros y licores incentivaría el comercio ilegal, advierte la SNI | Informative |
| El Comercio | 4 May 2018 | Spanish | Aumento avisado | Informative |
| Perú 21 Online | 4 May 2018 | Spanish | Bebidas azucaradas y alcohol pagarían mayores impuestos | Informative |
| Gestión Online | 4 May 2018 | Spanish | Alza de ISC a bebidas azucaradas tendrá impacto muy rápido en recaudación | Informative |
| Gestión Online | 5 May 2018 | Spanish | Tendencia mundial apunta a gravar bebidas azucaradas , sostiene Alonso Segura | Informative |
| El Comercio | 6 May 2018 | Spanish | Pulsoeconómico | Informative |
| Gestión Online | 6 May 2018 | Spanish | Alimentación Saludable: Gobierno reafirma que uso de octógonos es más claro que semáforo | Informative |
| Gestión Online | 7 May 2018 | Spanish | Minsa a favor de aplicar ISC para bebidas azucaradas | Informative |
| Gestión Online | 7 May 2018 | Spanish | Editorial: Cuidado con los detalles | Opinion |
| Esmerk Latin American News | 7 May 2018 | English | Peru: Pessah supports ISC tax initiative for sugary drinks | Informative |
| Gestión Online | 9 May 2018 | Spanish | Recaudación por ISC a combustibles cae desde el 2016 | Informative |

| Media Source | Date | Language | Title | Type of news (Informative, Opinion, Interview, Law) |
|---|---|---|---|---|
| Gestión Online | 9 May 2018 | Spanish | Empresas de bebidas responden al Gobierno: el ISC se paga desde el año 1999 | Informative |
| Gestión Online | 9 May 2018 | Spanish | Bodegueros de Lima estiman que sus ventas crecerán en 2018, pese a desaceleración económica | Informative |
| Gestión Online | 9 May 2018 | Spanish | Tuesta: “Obesidad afecta la productividad e impacta en 1% del PBI” | Informative |
| Reuters - Noticias Latinoamericanas | 9 May 2018 | Spanish | Perú elevará impuestos a productos que afectan a la salud pese a oposición de industria | Informative |
| El Comercio | 10 May 2018 | Spanish | El MEF confirma que se aumentará el ISC a las bebidas azucaradas | Informative |
| Reuters - Noticias Latinoamericanas | 10 May 2018 | Spanish | RPT-Perú elevará impuestos a productos que afectan a la salud pese a oposición de industria | Informative |
| ValorFuturo | 10 May 2018 | Spanish | MEF aprueba tasa de 25% en ISC a bebidas azucaradas ; bebidas con menor azúcar mantiene ISC en 17% | Informative |
| Gestión Online | 10 May 2018 | Spanish | MEF sube impuesto a bebidas alcohólicas para reducir su consumo | Informative |
| Gestión Online | 10 May 2018 | Spanish | MEF aprueba tasa de 25% en ISC a las bebidas azucaradas | Informative |
| Esmerk Latin American News | 10 May 2018 | English | Peru: Sugary drinks will pay higher ISC tax | Informative |
| ValorFuturo | 10 May 2018 | Spanish | MEF sube ISC de productos que más afectan a la salud y el ambiente; bebidas alcohólicas +35% | Informative |
| Perú 21 Online | 10 May 2018 | Spanish | MEF subió el impuesto a las gaseosas , licores, cigarros , combustibles y vehículos | Informative |
| Gestión Online | 10 May 2018 | Spanish | MEF: ISC a los cigarrillos sube a S/ 0.27 por cada uno | Informative |
| Reuters - Noticias Latinoamericanas | 10 May 2018 | Spanish | Perú sube impuesto a bebidas azucaradas y alcohólicas , cigarrillos y autos usados | Informative |
| dpa Servicio Internacional en Español | 10 May 2018 | Spanish | Perú sube impuesto a bebidas con alto contenido de azúcar y alcohol | Informative |
| dpa Servicio Internacional en Español | 10 May 2018 | Spanish | RPT RPT Perú sube impuesto a bebidas con alto contenido de azúcar y alcohol (dpa repite en economía esta nota emitida previamente en diversos) | Informative |
| Gestión Online | 10 May 2018 | Spanish | SNI sobre alza de ISC a cigarrillos : no bajará el consumo y crecerá el contrabando | Informative |
| Gestión Online | 10 May 2018 | Spanish | Bodegas afirman que sus ventas caerían 30% tras aumento en el ISC | Informative |
| Gestión Online | 10 May 2018 | Spanish | MEF: ISC a los cigarrillos sube a S/ 0.27 por cada unidad | Informative |

| Media Source | Date | Language | Title | Type of news (Informative, Opinion, Interview, Law) |
|---|---|---|---|---|
| Agencia EFE - Servicio Internacional | 10 May 2018 | Spanish | Perú sube impuestos a los productos que afectan a la salud y al medioambiente; PERÚ IMPUESTOS | Informative |
| ValorFuturo | 10 May 2018 | Spanish | Pdte Vizcarra: Alza del ISC a bebidas azucaradas busca cuidar salud y subir recaudación en Perú | Informative |
| Gestión Online | 10 May 2018 | Spanish | Alza en el ISC : El mayor impacto se verá en el transporte, sostiene Casas | Informative |
| ValorFuturo | 10 May 2018 | Spanish | Minam: Los combustibles más limpios le costarán menos a la población peruana tras alza de ISC | Informative |
| Agência CMA Latam | 10 May 2018 | Spanish | PERÚ: Gobierno sube a 25% tasa del ISC para bebidas azucaradas | Informative |
| Perú 21 Online | 10 May 2018 | Spanish | ¡Atentos! MEF subió impuesto a las gaseosas , licores, cigarros , combustibles y vehículos | Informative |
| Gestión Online | 10 May 2018 | Spanish | MEF a cerveceros artesanales: El ISC no es para reducir la competencia | Informative |
| Gestión Online | 10 May 2018 | Spanish | Cerveceros artesanales afirman que serán los más afectados con cambios al ISC | Informative |
| Gestión Online | 10 May 2018 | Spanish | Fabricantes de gaseosas : Alza en carga tributaria es una medida arbitraria y no transparente | Informative |
| Gestión Online | 10 May 2018 | Spanish | CCL: 30% de bebidas alcohólicas que se consumen en Perú son informales | Informative |
| Gestión Online | 10 May 2018 | Spanish | Alza al ISC : ¿Problema ambiental o aumento en la recaudación? | Informative |
| Gestión Online | 10 May 2018 | Spanish | Vizcarra defiende impuesto a bebidas azucaradas : "Estamos cambiando el ISC a productos que hacen daño" | Informative |
| Agencia EFE - Servicio Efeagro | 11 May 2018 | Spanish | Perú sube impuesto a tabaco , alcohol , carburantes, autos y bebidas azucaradas ; PERÚ IMPUESTOS | Informative |
| El Comercio | 11 May 2018 | Spanish | Impuesto con respuestas | Informative |
| Perú 21 Online | 11 May 2018 | Spanish | ¿Se Pusieron los Pantalones? | Opinion |
| Gestión Online | 11 May 2018 | Spanish | SNI: Precios de licores con mayor grado de concentración de alcohol subirán 20% | Informative |

| Media Source | Date | Language | Title | Type of news (Informative, Opinion, Interview, Law) |
|---|---|---|---|---|
| Gestión Online | 11 May 2018 | Spanish | Vizcarra afirma que alza del ISC tendrá un impacto mínimo: "creo que se justifica" | Informative |
| Esmerk Latin American News | 11 May 2018 | English | Peru: Higher ISC tax on alcohol will boost informal market | Informative |
| Esmerk Latin American News | 11 May 2018 | English | Peru: Higher ISC tax will hit alcohol market | Informative |
| Esmerk Latin American News | 11 May 2018 | English | Peru: ISC tax hike will affect drinks with artificial sweeteners too | Informative |
| Esmerk Latin American News | 11 May 2018 | English | Peru: Abresa warns of a 10% increase in cost of sugary drinks | Informative |
| Gestión Online | 11 May 2018 | Spanish | MEF a cerveceros artesanales: El ISC no es para reducir la competencia | Informative |
| Gestión Online | 11 May 2018 | Spanish | Fabricantes de gaseosas : Alza en carga tributaria es una medida arbitraria y no transparente | Informative |
| Gestión Online | 11 May 2018 | Spanish | Backus prevé "alto impacto" por alza del ISC a cervezas | Informative |
| ValorFuturo | 11 May 2018 | Spanish | SNI: Precios de licores con mayor grado de concentración de alcohol subirán 20% en Perú | Informative |
| Perú 21 Online | 11 May 2018 | Spanish | Asociación de fabricantes de gaseosas considera arbitrario el aumento del impuesto a estas bebidas | Informative |
| Gestión Online | 11 May 2018 | Spanish | Confiep “duda” de los impactos que tendrá el aumento del ISC | Informative |
| El Comercio | 12 May 2018 | Spanish | Alcoholismo tributario | Opinion |
| El Comercio | 12 May 2018 | Spanish | La subida del ISC a las bebidas azucaradas | Opinion |
| El Mercurio | 12 May 2018 | Spanish | Automotoras chilenas complicadas en Perú por fuerte alza de impuesto a importación | Informative |
| Perú 21 Online | 12 May 2018 | Spanish | Impuestos no afectarán precios en más de 0.3% | Informative |
| Perú 21 Online | 12 May 2018 | Spanish | El que chupa contribuye | Opinion |
| El Comercio | 13 May 2018 | Spanish | Pulsoeconómico | Informative |
| El Comercio | 13 May 2018 | Spanish | ?La política económica no se hace de forma plebiscitaria? | Opinion |
| El Comercio | 14 May 2018 | Spanish | Dos noticias de gran impacto en la salud pública | Opinion |

| Media Source | Date | Language | Title | Type of news (Informative, Opinion, Interview, Law) |
|---|---|---|---|---|
| Perú 21 Online | 14 May 2018 | Spanish | Luis Galarreta dijo que aumento al ISC fue un "tema de recaudación" | Informative |
| Perú 21 Online | 14 May 2018 | Spanish | Datum: Más del 60% a favor de subir impuestos a cigarros y licores | Informative |
| Gestión Online | 14 May 2018 | Spanish | Editorial: Renace el optimismo | Informative |
| Esmerk Latin American News | 14 May 2018 | English | Peru: Pisco will not pay higher ISC tax | Informative |
| Esmerk Latin American News | 14 May 2018 | English | Peru: Backus questions ISC tax hike | Informative |
| ValorFuturo | 14 May 2018 | Spanish | Recaudación de ISC a combustibles, gaseosas y cervezas cae 5% en lo que va del año en Perú -BCRP | Informative |
| Gestión Online | 14 May 2018 | Spanish | Recaudación de ISC crece casi 6% en abril pero cae 5% en acumulado | Informative |
| Esmerk Latin American News | 14 May 2018 | English | Peru: Over 50% of Peruvians back tax increase for sugary drinks | Informative |
| Esmerk Latin American News | 14 May 2018 | English | Peru: Ministry of Health should draw up index of harmfulness | Informative |
| Gestión Online | 15 May 2018 | Spanish | Scotiabank: Inflación retornaría al rango meta en mayo | Informative |
| Perú 21 Online | 15 May 2018 | Spanish | César Villanueva: "No hemos entrado para ser populares" | Informative |
| Gestión Online | 15 May 2018 | Spanish | Editorial: Alza selectiva | Opinion |
| El Comercio | 16 May 2018 | Spanish | Impopular pero correcto | Opinion |
| El Comercio | 16 May 2018 | Spanish | Cambios selectivos | Opinion |
| Gestión Online | 16 May 2018 | Spanish | Minam busca que entidades financieras generen créditos verdes para vehículos | Interview |
| Reuters - Noticias Latinoamericanas | 17 May 2018 | Spanish | Ingresos tributarios de Perú suben 40 pct interanual en abril | Informative |
| SentidoComun.com.mx | 17 May 2018 | Spanish | Arca Continental pospone compra resto Lindley tras impuesto refrescos Perú | Informative |
| El Comercio | 18 May 2018 | Spanish | Cambios en el impuesto selectivo | Opinion |
| Gestión Online | 18 May 2018 | Spanish | AC Bebidas pospone compra de acciones de Peru Beverage en Lindley por aumento del ISC | Informative |
| ValorFuturo | 18 May 2018 | Spanish | Arca Continental pospone compra de acciones de Peru Beverage en Lindley por aumento de ISC | Informative |
| El Comercio | 20 May 2018 | Spanish | ISC : una opinión más para el debate | Opinion |

| **Media Source** | **Date** | **Language** | **Title** | **Type of news (Informative, Opinion, Interview, Law)** |
|---|---|---|---|---|
| El Comercio | 20 May 2018 | Spanish | ¿A su salud? | Opinion |
| El Financiero | 21 May 2018 | Spanish | Afectaría impuesto en Perú a volumen de AC | Informative |
| Gestión Online | 21 May 2018 | Spanish | ISC es una mala medida, aislada y contradictoria, afirma Sandro Fuentes | Informative |
| Esmerk Latin American News | 21 May 2018 | English | Peru: Coca-Cola increases prices due to ISC tax hike | Informative |
| El Comercio | 24 May 2018 | Spanish | Impuestos doble cara | Informative |
| Gestión Online | 24 May 2018 | Spanish | Colat: Industria tabacalera aumentó sus precios en 70% sin que se suba el ISC | Informative |
| Perú 21 Online | 24 May 2018 | Spanish | Martín Vizcarra asegura que alza en el precio de los combustibles no se debe al ISC | Informative |
| Gestión Online | 24 May 2018 | Spanish | El Cusco acata paralización por incremento del impuesto a los combustibles | Informative |
| El Comercio | 28 May 2018 | Spanish | Budweiserse relanza y crece en distribución | Informative |
| El Comercio | 28 May 2018 | Spanish | Perú, arriba en diversidad de vinos de alta gama en Latinoamérica | Informative |
| Perú 21 Online | 28 May 2018 | Spanish | Entre chihuahuas y dobermans | Opinion |
| Gestión Online | 28 May 2018 | Spanish | Editorial: Falta de atención | Informative |
| Semana Económica | 28 May 2018 | Spanish | OCDE: "Tiene sentido ampliar la base tributaria y que haya más peruanos pagando" | Interview |
| El Comercio | 29 May 2018 | Spanish | Retos tributarios | Informative |
| Gestión Online | 30 May 2018 | Spanish | Julio Velarde: "Se ha exagerado mucho con el alza de precios de los combustibles" | Informative |
| BBC Monitoring Americas | 31 May 2018 | English | Bolivia, Peru media highlights 30 May 2018 | Informative |
| El Comercio | 1 June 2018 | Spanish | Una inflación que aún no preocupa | Informative |
| El Comercio | 4 June 2018 | Spanish | Tributos y octanos | Opinion |
| Gestión Online | 4 June 2018 | Spanish | Editorial: Impacto atenuado | Informative |

| Media Source | Date | Language | Title | Type of news (Informative, Opinion, Interview, Law) |
|---|---|---|---|---|
| Semana Económica | 4 June 2018 | Spanish | <!DOCTYPE html PUBLIC "-//W3C//DTD XHTML 1.0 Transitional//EN" "http://www.w3.org/TR/xhtml1/DTD/xhtml1-transitional.dtd"> <html xmlns="http://www.w3.org/1999/xhtml"> <head> <title></title> </head> <body> David Tuesta renunció al Ministerio de Economía </body> </html> | Informative |
| Europa Press - Servicio Internacional | 4 June 2018 | Spanish | Perú.- Dimite el ministro de Economía de Perú dos meses después de acceder al cargo | Informative |
| Agencia EFE - Servicio Internacional | 4 June 2018 | Spanish | Renuncia el ministro de Economía y Finanzas de Perú, David Tuesta; PERÚ GOBIERNO | Informative |
| CE NoticiasFinancieras | 5 June 2018 | Spanish | David Tuesta renunció al Ministerio de Economía | Informative |
| Europa Press - Servicio Internacional | 5 June 2018 | Spanish | Perú.- Vizcarra acepta la dimisión de Tuesta y asegura estar reformando el Gabinete | Informative |
| Reuters - Noticias Latinoamericanas | 5 June 2018 | Spanish | ACTUALIZA 3-Presidente Perú confirma renuncia de ministro Economía, buscará mejor recaudación | Informative |
| Agencia EFE - Servicio Internacional | 5 June 2018 | Spanish | El presidente de Perú acepta la renuncia del ministro de Economía; PERÚ GOBIERNO | Informative |
| Agencia Mexicana de Noticias, NOTIMEX | 5 June 2018 | Spanish | Perú adelanta nueva política financiera tras renuncia de ministro | Informative |
| El Comercio | 5 June 2018 | Spanish | Renuncia en el MEF | Informative |
| Reuters - Noticias Latinoamericanas | 5 June 2018 | Spanish | RPT-ACTUALIZA 3-Presidente Perú confirma renuncia de ministro Economía, buscará mejor recaudación | Informative |
| CE NoticiasFinancieras | 5 June 2018 | Spanish | Presidente de Perú confirma renuncia de ministro de Economía | Informative |
| Gestión Online | 5 June 2018 | Spanish | Salida de Tuesta: ¿Qué deja y qué retos asume el nuevo ministro de Economía? | Informative |
| ValorFuturo | 5 June 2018 | Spanish | Alza de ISC a combustibles más contaminantes en Perú es una medida acertada -IPE | Informative |
| Reuters - Noticias Latinoamericanas | 5 June 2018 | Spanish | ACTUALIZA 3-Presidente Perú considera tres nombres para ministro Economía, no cambiará política | Informative |
| CE NoticiasFinancieras | 5 June 2018 | English | David Tuesta resigned from the Ministry of Economy | Informative |
| El País - Nacional | 6 June 2018 | | Dimite en Perú el ministro de Economía tras dos meses en el cargo | Informative |
| Gestión Online | 7 June 2018 | Spanish | Primavera fugaz: sube 25 puntos la desaprobación del presidente Vizcarra | Informative |

| **Media Source** | **Date** | **Language** | **Title** | **Type of news (Informative, Opinion, Interview, Law)** |
|---|---|---|---|---|
| Europa Press - Servicio Internacional | 7 June 2018 | Spanish | Perú.- Carlos Oliva jura el cargo como nuevo ministro de Economía de Perú | Informative |
| Diario Financiero | 8 June 2018 | Spanish | Perú no sale de la crisis: asume el sexto jefe de Economía desde 2016 y crece rechazo a Vizcarra | Informative |
| IHS Global Insight Daily Analysis | 8 June 2018 | English | Appointment of new Peruvian finance minister reduces risk of tax increases and protests; fiscal-capture efforts to continue | Informative |
| Perú 21 Online | 10 June 2018 | Spanish | Elmer Cuba: “Tienen la mesa servida, no la echen a perder” | Interview |
| Europa Press - Notiamerica | 10 June 2018 | Spanish | Perú.- El índice de aprobación de Vizcarra cae 15 puntos en el marco de las protestas por la subida de impuestos | Informative |
| Perú 21 Online | 11 June 2018 | Spanish | Nueve de cada diez peruanos sienten que el ISC elevó precios | Informative |
| Gestión Online | 13 June 2018 | Spanish | Asociación de Bodegueros: Estos son los impactos del aumento del ISC | Informative |
| El Comercio | 13 June 2018 | Spanish | El impuesto de la discordia | Informative |
| Reuters - Noticias Latinoamericanas | 14 June 2018 | Spanish | ACTUALIZA 1-Perú no dará "marcha atrás" en aumento del impuesto selectivo al consumo : ministro | Informative |
| People’s Daily Online | 15 June 2018 | Spanish | Política económica busca bienestar social en Perú | Informative |
| Perú 21 Online | 15 June 2018 | Spanish | Aprueban facultades tributarias y MEF aclara que alza del ISC continúa | Informative |
| Gestión Online | 17 June 2018 | Spanish | Sunat: Recaudación del IGV interno aumentó 18% en mayo por mayor demanda interna | Informative |
| Perú 21 Online | 18 June 2018 | Spanish | Apoyo: PBI crecería casi 4% en el segundo trimestre | Informative |
| ValorFuturo | 18 June 2018 | Spanish | Ingresos tributarios de Perú crecieron 22% a PEN 8.199 MM en May 2018/17; IGV Total +13,8% | Informative |
| Gestión Online | 1 July 2018 | Spanish | Inflación en Lima fue de 0.33% en junio, impactada por alza del ISC | Informative |
| Perú 21 Online | 1 July 2018 | Spanish | Precios en Lima crecieron 0.33% en junio, según INEI | Informative |
| Gestión Online | 2 July 2018 | Spanish | BCP: Inflación cerraría en 2.5% este año por alza del petróleo y commodities agrícolas | Informative |
| ValorFuturo | 3 July 2018 | Spanish | Scotiabank: Inflación de julio sería similar a la de junio en Perú; mantiene 2% para fin de 2018 | Informative |
| ValorFuturo | 6 July 2018 | Spanish | Inflación anual en Perú vuelve al rango meta del BCRP al pasar de 0,93% a 1,43% en Jun | Informative |

| **Media Source** | **Date** | **Language** | **Title** | **Type of news (Informative, Opinion, Interview, Law)** |
|---|---|---|---|---|
| Esmerk Latin American News | 9 July 2018 | English | Peru: ISM increases prices by around 10% due to tax hike | Informative |
| Perú 21 Online | 10 July 2018 | Spanish | Impacto del aumento en el ISC | Opinion |
| ValorFuturo | 16 July 2018 | Spanish | Ingresos por ISC en Perú sumaron PEN 753 MM en Jun 2018/17 y crecieron 55,2% por alza de tasas -BCRP | Informative |
| Semana Económica | 24 July 2018 | Spanish | KWP: “Se viene una etapa finalmente buena para el consumo en el Perú” | Informative |
| El Comercio | 28 July 2018 | Spanish | Algunos de los avances económicos de la era Vizcarra | Informative |
| El Comercio | 2 August 2018 | Spanish | Ventas de empresas top crecen a doble dígito | Informative |
| Gestión Online | 3 August 2018 | Spanish | Perú lidera el consumo per cápita de alcohol ilegal en la región | Informative |
| El Comercio | 10 August 2018 | Spanish | El ISC no frenó el consumo de bienes gravados | Informative |
| Perú 21 Online | 13 August 2018 | Spanish | Moody's: consumo privado crecerá a 3.6% hacia 2019, pese a impacto del ISC | Informative |
| Reforma.com | 19 August 2018 | Spanish | Afecta a refresqueras impuesto al azúcar | Informative |
| CE NoticiasFinancieras | 20 August 2018 | Spanish | Afectan a refresqueras impuestos al azúcar | Informative |
| Perú 21 Online | 1 September 2018 | Spanish | INEI: Precios en Lima subieron 0.13% durante agosto | Informative |
| El Financiero | 2 September 2018 | Spanish | AC Bebidas reanudó negociaciones para incrementar participación en Lindley | Informative |
| SentidoComun.com.mx | 3 September 2018 | Spanish | Arca Continental reinicia intento por comprar resto de peruana Lindley | Informative |
| El Comercio | 10 September 2018 | Spanish | Pernod Ricard cambia estrategia ante subida del ISC | Informative |
| El Financiero | 26 September 2018 | Spanish | Arca concreta compra de acciones de Corporación Lindley | Informative |
| ValorFuturo | 2 October 2018 | Spanish | Sunat: Incremento del ISC a bebidas alcohólicas en Perú no incentiva el contrabando | Informative |
| Perú 21 Online | 3 October 2018 | Spanish | Alcohol adulterado vale 25% menos que el legal | Informative |
| Andina Agencia Peruana de Noticias | 3 October 2018 | Spanish | Mayor ISC a bebidas alcohólicas no incentivó el contrabando | Informative |

| Media Source | Date | Language | Title | Type of news (Informative, Opinion, Interview, Law) |
|---|---|---|---|---|
| ValorFuturo | 4 October 2018 | Spanish | Recaudación de ISC en Perú se recupera y crece 0,6% a PEN 4.379,6 MM en primeros 8 meses de 2018 | Informative |
| El Comercio | 15 October 2018 | Spanish | Grupo Campari relanza Ricadonna y amplía portafolio | Informative |
| Perú 21 Online | 24 October 2018 | Spanish | Muy peruano: Empiezan los Felipillos... | Opinion |
| Gestión Online | 28 October 2018 | Spanish | SNI: Producción de gaseosas cae 13.7% tras alza del ISC | Informative |
| El Comercio | 29 October 2018 | Spanish | CBC apuesta por retornables para crecer con Pepsi | Informative |
| Gestión Online | 4 November 2018 | Spanish | Licorerías Almendariz: "Ha sido un año duro para el mercado por alza del ISC " | Informative |
| Esmerk Latin American News | 5 November 2018 | English | Peru: Non- alcoholic drinks industry focuses on healthier products | Informative |
| El Comercio | 7 November 2018 | Spanish | ISC favorece a licores con más grado de alcohol | Informative |
| El Comercio | 3 December 2018 | Spanish | salto pendienteNo despega el consumo | Informative |
| El Comercio | 6 December 2018 | Spanish | Otras preguntas | Opinion |
| El Comercio | 28 December 2018 | Spanish | Medidas con expectativas excesivas | Opinion |
| Gestión Online | 28 December 2018 | Spanish | Una de cada cuatro bebidas alcohólicas comercializadas es ilegal | Informative |
| Gestión Online | 1 January 2019 | Spanish | Precios al Consumidor a Nivel Nacional aumentan 0.19% en diciembre del 2018 | Informative |
| Gestión Online | 1 January 2019 | Spanish | Inflación cerró el 2018 en 2.19% luego de reportar un alza de 0.18% en diciembre | Informative |
| El Comercio | 2 January 2019 | Spanish | Inflación sin alimentos supera 2% | Informative |
| Gestión Online | 18 January 2019 | Spanish | Sunat: Recaudación tributaria subió 13.8% el 2018, luego de tres años de caídas | Informative |
| Perú 21 Online | 18 January 2019 | Spanish | Recaudación tributaria creció 13.8% en 2018 después de tres años de caídas | Informative |
| Esmerk Latin American News | 21 January 2019 | English | Peru: Illegal alcohol sales up by 5% since ISC tax hike | Informative |
| El Comercio | 21 January 2019 | Spanish | CCL: Licores informales ganan terreno | Informative |

| Media Source | Date | Language | Title | Type of news (Informative, Opinion, Interview, Law) |
|---|---|---|---|---|
| El Comercio | 28 January 2019 | Spanish | Equilibrando la presión | Informative |
| Gestión Online | 28 January 2019 | Spanish | BCP: Déficit fiscal descendería a 2.3% del PBI en el 2019 | Informative |
| Semana Económica | 21 February 2019 | Spanish | AC Lindley apunta a la recuperación tras caída en ingresos y ganancias en el 2018 | Informative |
| Esmerk Latin American News | 22 February 2019 | English | Peru: Premium beers boost Backus sales in 2018 | Informative |
| CE NoticiasFinancieras | 27 February 2019 | English | Grupo AJE launched super fruits drinks from the Amazon | Informative |
| La República (Peru) | 7 March 2019 | Spanish | Producción de agua embotellada crecería 7,9% este año | Informative |
| El Comercio | 9 March 2019 | Spanish | Jefes del Gabinete duran en el cargo menos de un año en promedio | Informative |
| Gestión Online | 19 March 2019 | Spanish | Agua embotellada: ¿Se avecina guerra de precios en este mercado? | Informative |
| Gestión Online | 19 March 2019 | Spanish | Los ministros que Martín Vizcarra perdió en su primer año de Gobierno | Informative |
| Gestión Online | 20 March 2019 | Spanish | Naturale: “El consumo de bebidas saludables representa el 14% del mercado en Perú” | Informative |
| La República (Peru) | 25 March 2019 | Spanish | Baja recaudación de Perú pone en riesgo su desarrollo | Informative |
| La República (Peru) | 25 March 2019 | Spanish | Qué hacen los congresistas de Arequipa en semana de representación | Informative |
| Semana Económica | 15 April 2019 | Spanish | Conveniencia y sofisticación: los drivers de crecimiento de las bebidas espirituosas | Informative |
| Semana Económica | 25 April 2019 | Spanish | Cartavio sigue creciendo pese a alza de ISC y evalúa ingresar a nueva categoría | Informative |
| Andina Agencia Peruana de Noticias | 21 May 2019 | Spanish | Recaudación en regiones del sur aumentó 13.5% en 2018 | Informative |
| Esmerk Latin American News | 27 May 2019 | English | Peru: Backus reveals results, plans and strategies | Informative |
| Andina Agencia Peruana de Noticias | 4 June 2019 | Spanish | La recaudación por ISC sumó S/ 2,705 millones | Informative |
| Diario Oficial de Peru | 15 June 2019 | Spanish | DECRETO SUPREMO N° 181-2019-EF | Law |

| Media Source | Date | Language | Title | Type of news (Informative, Opinion, Interview, Law) |
|---|---|---|---|---|
| La República (Peru) | 15 June 2019 | Spanish | Ejecutivo modificó el Impuesto Selectivo al Consumo e Impuesto a la Renta | Informative |
| La República (Peru) | 16 June 2019 | Spanish | Ejecutivo aplicó cambios al Impuesto Selectivo al Consumo | Informative |
| Gestión Online | 17 June 2019 | Spanish | Precios de cervezas populares podrían verse afectadas | Informative |
| La República (Peru) | 17 June 2019 | Spanish | MEF: Cambios en el ISC no elevará el costo de las cervezas | Informative |
| Esmerk Latin American News | 17 June 2019 | English | Peru: ISC tax lowered for low-sugar drinks and new cars | Informative |
| Semana Económica | 18 June 2019 | Spanish | Importadores podrán recuperar pago del ISC si autos nuevos son convertidos a gas | Informative |
| La República (Peru) | 18 June 2019 | Spanish | Impuesto a la cerveza ahora será por litro y no sobre el precio | Informative |
| El Comercio | 18 June 2019 | Spanish | MEF descarta que ajuste al ISC eleve precio de la cerveza | Informative |
| Andina Agencia Peruana de Noticias | 18 June 2019 | Spanish | Prevén menor demanda de vehículos usados importados | Informative |
| La República (Peru) | 18 June 2019 | Spanish | Impuesto a la cerveza : ¿subirá o no el precio? | Informative |
| Gestión Online | 20 June 2019 | Spanish | Sube consumo de whisky premium en Perú | Informative |
| Esmerk Latin American News | 21 June 2019 | English | Peru: AJE criticises ISC tax adjustment for beers | Informative |
| Perú 21 Online | 25 June 2019 | Spanish | SNI cuestiona aplicación del ISC a industria vitivinícola y afirma que es "discriminador y muy alto" | Informative |
| Gestión Online | 25 June 2019 | Spanish | SNI pide cambios al ISC al vino por ser uno de los más altos en relación a otros países | Informative |
| La República (Peru) | 26 June 2019 | Spanish | Backus aplica incremento de 10% en el precio de sus cervezas por alza del ISC | Informative |
| Perú 21 Online | 25 June 2019 | Spanish | Precios de las cervezas de Backus suben un 10%, excepto en presentaciones de un litro | Informative |
| Esmerk Latin American News | 26 June 2019 | English | Peru: Wine industry asks for ISC tax change to be reviewed | Informative |

| **Media Source** | **Date** | **Language** | **Title** | **Type of news (Informative, Opinion, Interview, Law)** |
|---|---|---|---|---|
| Esmerk Latin American News | 26 June 2019 | English | Peru: Backus increases beer prices by 10% due to ISC tax hike | Informative |
| La República (Peru) | 27 June 2019 | Spanish | La primera etapa del megapuerto de Chancay casi triplicará su inversiónAspec: Ausencia de Ley de Fusiones genera alza de preciosComercio creció 2,98% en abril por ventas al por mayor y menorUso de tarjetas de crédito es alto entre peruanos | Informative |
| Semana Económica | 27 June 2019 | Spanish | Ejecutivo no observó ley de fortalecimiento de Zofratacna pese a reparos del MEF | Informative |
| La República (Peru) | 29 June 2019 | Spanish | Cervezas de Backus suben de precio en 10% | Informative |
| La República (Peru) | 30 June 2019 | Spanish | Externalidades e impuestos | Opinion |
| La República (Peru) | 1 July 2019 | Spanish | Inflación fue negativa en junio: precios cayeron 0,09% | Informative |
| La República (Peru) | 1 July 2019 | Spanish | INEI: Precio de la cerveza subió 1,1% en junio | Informative |
| Perú 21 Online | 1 July 2019 | Spanish | Inflación anualizada bajó a 2.29% en junio | Informative |
| La República (Peru) | 1 July 2019 | Spanish | Oliva: “No tendría por qué haberse incrementado el precio de la cerveza ” | Informative |
| La República (Peru) | 2 July 2019 | Spanish | Oliva: “Hay condiciones para darle la licencia a Tía María” | Informative |
| Gestión Online | 3 July 2019 | Spanish | Congreso promulga ley de fortalecimiento de zona franca de Tacna pese a oposición del MEF | Informative |
| La República (Peru) | 3 July 2019 | Spanish | CCL pide eliminar facultad del Ejecutivo para modificar el ISC | Informative |
| El Comercio | 9 July 2019 | Spanish | Cambios en ISC generarían una recaudación adicional al año de S/530 mlls. | Informative |
| La República (Peru) | 9 July 2019 | Spanish | Cerveza \| Grupo AJE: modificación del ISC a la cerveza favorece el monopolio | Informative |
| Gestión Online | 12 July 2019 | Spanish | COMUNICADO A LA OPINIÓN PÚBLICA | Informative |
| El Comercio | 15 July 2019 | Spanish | Pernod Ricard crece con Beefeater Pink y Chivas XV | Informative |
| Gestión Online | 24 July 2019 | Spanish | Gobierno evalúa que productos con ‘grasas trans’ paguen impuesto | Informative |
| El Comercio | 27 July 2019 | Spanish | ?Avanzar reformas requiere sacrificios de popularidad? | Interview |
| La República (Peru) | 1 August 2019 | Spanish | Alza en precio de las cervezas y bebidas alcohólicas elevó la inflación de julio a 0,23% | Informative |

| Media Source | Date | Language | Title | Type of news (Informative, Opinion, Interview, Law) |
|---|---|---|---|---|
| Esmerk Latin American News | 2 August 2019 | English | Peru: Backus beer sales increase | Informative |
| El Comercio | 5 August 2019 | Spanish | Generade se potencia y entrará a Lima a fin de año | Informative |
| La República (Peru) | 17 August 2019 | Spanish | Recaudación de impuestos se redujo 2,9% en julio | Informative |
| Esmerk Latin American News | 21 August 2019 | English | Peru: Households without children buy the most bottled water | Informative |
| CE NoticiasFinancieras | 14 September 2019 | Spanish | CE Noticias Financieras Spanish | Informative |
| El Comercio | 18 September 2019 | Spanish | Ministerios buscan regular mercado de cigarros electrónicos | Informative |
| La República (Peru) | 27 September 2019 | Spanish | INEI: Sector comercio creció 3,19% en julio | Informative |
| El Comercio | 14 October 2019 | Spanish | ?Litrazo?, la apuesta de Pilsen para volverse más accesible | Informative |
| Diario Oficial de Peru | 31 October 2019 | Spanish | RESOLUCION N° 217-2019/SUNAT | Law |
| Andina Agencia Peruana de Noticias | 17 November 2019 | Spanish | Recaudación del impuesto a la renta creció 16.3% | Informative |
| El Comercio | 24 November 2019 | Spanish | ?La promesa del presidente Vizcarra de ?evaluar? el incremento del ISC a los combustibles ante las protestas del interior del pa | Informative |
| El Comercio | 6 January 2020 | Spanish | Golden, la nueva apuesta de Backus | Informative |
| La República (Peru) | 6 January 2020 | Spanish | Sunat recaudó 110.768 millones de soles en tributos netos durante el 2019 | Informative |
| Euromonitor Sector Capsules | 6 January 2020 | English | Euromonitor Sector Capsule; Juice in Peru | Informative |
| Andina Agencia Peruana de Noticias | 7 January 2020 | Spanish | Medidas de Sunat permiten incrementar la recaudación | Informative |
| El Comercio | 13 January 2020 | Spanish | Las cartas de Jack Daniel?sen el Perú | Informative |
| Diario Oficial de Peru | 24 January 2020 | Spanish | RESOLUCION MINISTERIAL N° 034 -2020-EF/15 | Law |
| La República (Peru) | 25 January 2020 | Spanish | Precio del pisco, cigarros y algunas bebidas alcohólicas subirán desde el domingo ¿Por qué? [infografía] | Informative |
| La República (Peru) | 25 January 2020 | Spanish | Impuesto del pisco, cigarros y algunas bebidas alcohólicas suben desde mañana | Informative |



Zuleta M, *et al. BMJ Glob Health* 2023; 8:e012024. doi: 10.1136/bmjgh-2023-012024

| Media Source | Date | Language | Title | Type of news (Informative, Opinion, Interview, Law) |
|---|---|---|---|---|
| Andina Agencia Peruana de Noticias | 25 January 2020 | Spanish | Descartan elevar tasas del ISC por actualización | Informative |
| La República (Peru) | 25 January 2020 | Spanish | Ministra Alva explica ajuste del impuesto al cigarro , pisco y bebidas alcohólicas | Informative |
| Diario Oficial de Peru | 25 January 2020 | Spanish | RESOLUCION MINISTERIAL N° 042-2020-EF/43 | Law |
| Esmerk Latin American News | 25 January 2020 | English | Peru: ISC tax rises due to inflation [amended] | Informative |
| El Comercio | 26 January 2020 | Spanish | Pulsoeconómico | Informative |
| Esmerk Latin American News | 27 January 2020 | English | Peru: Rise in ISC tax may affect pisco consumption | Informative |
| El Comercio | 28 January 2020 | Spanish | Radar económico | Informative |
| Andina Agencia Peruana de Noticias | 1 February 2020 | Spanish | Precios al consumidor subió 0.05% en enero | Informative |
| El Comercio | 3 February 2020 | Spanish | Cerveza artesanal: Candelaria vuelvea la carga | Informative |
| La República (Peru) | 4 February 2020 | Spanish | Servicios digitales se incluirán en la canasta familiar | Informative |
| Agência CMA Latam | 4 February 2020 | Spanish | PERÚ: Indice de precios al consumidor sube a 1,89% en enero en 12 meses | Informative |
| La República (Peru) | 6 February 2020 | Spanish | Alza del ISC al cigarro y bebidas alcohólicas afecta a 500 mil bodegueros | Informative |
| El Comercio | 17 April 2020 | Spanish | ?La preocupación más grande es la cadena de valor? | Interview |
| El Comercio | 1 August 2020 | Spanish | Más del 20% de cervecerías artesanales en riesgo | Informative |
| Diario Oficial de Peru | 10 October 2020 | Spanish | RESOLUCION 000174-2020/SUNAT | Law |
| El Comercio | 30 November 2020 | Spanish | ?Tres a cuatro marcas de nuestro portafolio global entrarán el 2021? | Interview |
| La República (Peru) | 15 January 2021 | Spanish | MEF estaría evaluando elevar ISC para bebidas alcohólicas y cigarros | Informative |
| La República (Peru) | 24 January 2021 | Spanish | Waldo Mendoza: “La ministra de Salud tiene razón: estamos en una segunda ola” | Interview |
| La República (Peru) | 24 January 2021 | Spanish | MEF aumentará el ISC a la cerveza , cigarros y otros licores desde esta semana | Informative |

| Media Source | Date | Language | Title | Type of news (Informative, Opinion, Interview, Law) |
|---|---|---|---|---|
| La República (Peru) | 25 January 2021 | Spanish | MEF aumentará el ISC a cerveza , cigarros y otros licores desde esta semana | Informative |
| El Comercio | 25 January 2021 | Spanish | Elevarán monto para acceder a información bancaria | Informative |
| El Comercio | 26 January 2021 | Spanish | Gremios preocupados por alza del ISC | Informative |
| La República (Peru) | 26 January 2021 | Spanish | MEF aumenta el ISC al cigarro , cerveza , pisco y otros licores | Informative |
| Andina Agencia Peruana de Noticias | 26 January 2021 | Spanish | MEF eleva el ISC para cervezas , cigarrillos , pisco y otras bebidas | Informative |
| ValorFuturo | 26 January 2021 | Spanish | Actualización de ISC a cerveza , cigarro y licores permitirá recaudar PEN 100 millones más - MEF Perú | Informative |
| Diario Oficial de Peru | 26 January 2021 | Spanish | RESOLUCION MINISTERIAL N° 035-2021-EF/15 | Law |
| La República (Peru) | 27 January 2021 | Spanish | Alza del ISC al cigarro , pisco, cerveza y otros licores rige desde hoy | Informative |
| El Comercio | 27 January 2021 | Spanish | ADEX pide impulsar exportación del pisco ante cambios en ISC | Informative |
| Agência CMA Latam | 27 January 2021 | Spanish | PERÚ: Gob. actualiza impuesto para cerveza , tabaco y bebidas alchólicas | Informative |
| El Comercio | 1 February 2021 | Spanish | el aumento del isc en tiempos de una segunda ola | Opinion |
| El Comercio | 8 March 2021 | Spanish | Entre readaptarse o cerrar | Informative |
| CE NoticiasFinancieras | 14 March 2021 | English | Wine industry: how do Peru's most iconic brands cope with the second wave? | Informative |
| Andina Agencia Peruana de Noticias | 18 March 2021 | Spanish | Demandas de los transportistas son inaceptables, afirma ministro Waldo Mendoza | Informative |
| Andina Agencia Peruana de Noticias | 20 March 2021 | Spanish | Waldo Mendoza: Tenemos que concentrar recursos para la adquisición de vacunas | Informative |
| Andina Agencia Peruana de Noticias | 30 September 2021 | Spanish | Bebidas azucaradas : MEF modifica parámetros para Impuesto Selectivo al Consumo | Informative |
| La República (Peru) | 30 September 2021 | Spanish | Gaseosas , agua y cerveza sin alcohol que pasen del 5% de azúcar pagarán 25% de ISC | Informative |



Zuleta M, *et al. BMJ Glob Health* 2023; 8:e012024. doi: 10.1136/bmjgh-2023-012024

| Media Source | Date | Language | Title | Type of news (Informative, Opinion, Interview, Law) |
|---|---|---|---|---|
| CE NoticiasFinancieras | 30 September 2021 | English | Beverage companies' guild: Peru charges the highest ISC in the region | Informative |
| Diario Oficial de Peru | 30 September 2021 | Spanish | DECRETO SUPREMO N° 266-2021-EF | Law |
| La República (Peru) | 1 October 2021 | Spanish | Industria de bebidas garantiza continuidad de operaciones tras aumento del ISC | Informative |
| El Comercio | 1 October 2021 | Spanish | Modifican valla para aplicar el ISC | Informative |
| Andina Agencia Peruana de Noticias | 1 October 2021 | Spanish | Varían criterios del impuesto selectivo al consumo | Informative |
| Obesity & Diabetes Week | 11 October 2021 | English | Social Sciences; Sugar-sweetened beverages tax in Peru: A tax nested in history in the last two decades | Informative |
| La República (Peru) | 21 October 2021 | Spanish | ¿Qué significa ISC en Perú y qué productos afecta? | Informative |
| La República (Peru) | 21 October 2021 | Spanish | Consumo interno: sector bebidas fue el que más creció durante el segundo trimestre | Informative |
| wc | 9 November 2021 | | Perú consigue posicionarse con el Whiskey más premiado de Sudamérica | Informative |
| CE NoticiasFinancieras | 10 November 2021 | English | Non- alcoholic beer is gaining ground among young sportsmen and women | Informative |
| La República (Peru) | 1 December 2021 | Spanish | SNI solicitará un impuesto selectivo diferencial para la cerveza artesanal | Informative |
| Press Perú | 15 December 2021 | | Asovape: Los vapeadores pueden ayudar a salvar miles de vidas, pero aquí plantean ponerle impuestos altísimos | Informative |
| El Trome | 16 December 2021 | | Vendedores de vapeadores critican posible impuesto a sus productos | Informative |
| Press Perú | 12 January 2022 | | Bodegueros Preocupados Ante La Competencia Desleal Del Comercio Ilícito | Informative |
| El Tiempo | 14 January 2022 | Spanish | Por alza de Impuesto Selectivo aumentarían cifras del contrabando | Informative |
| La República (Peru) | 30 January 2022 | Spanish | MEF actualiza ISC de cigarrillos , pisco y bebidas alcohólicas con más de 20 grados de alcohol | Informative |
| Andina Agencia Peruana de Noticias | 30 January 2022 | Spanish | Actualizan el Impuesto Selectivo al Consumo de cigarrillos y bebidas alcohólicas | Informative |

| Media Source | Date | Language | Title | Type of news (Informative, Opinion, Interview, Law) |
|---|---|---|---|---|
| La República (Peru) | 30 January 2022 | Spanish | Cotización del dólar: ¿cuál es el tipo de cambio HOY, domingo 30 de enero? | Informative |
| Diario Oficial de Peru | 30 January 2022 | Spanish | RESOLUCION MINISTERIAL N° 021-2022-EF/15 | Law |
| CE NoticiasFinancieras | 31 January 2022 | English | BAT: ISC increase will encourage consumption of smuggled products | Informative |
| Andina Agencia Peruana de Noticias | 31 January 2022 | Spanish | MEF actualiza monto fijo del ISC a los cigarrillos y al pisco | Informative |
| La República (Peru) | 1 February 2022 | Spanish | BAT advierte que incrementos del ISC fomentan el consumo de cigarrillos de contrabando | Informative |



Zuleta M, *et al. BMJ Glob Health* 2023; 8:e012024. doi: 10.1136/bmjgh-2023-012024

Supplementary Material 3: Codebook

| **Codes**<br>**(B =In favour; C =Against)** | **Definition** |
|---|---|
| b01. F - Source of revenue for the government | Taxation as a source of income for the government. |
| b02. F - Reducing consumption | Taxation will help reduce the consumption of alcohol, sugar sweetened beverages and cigarettes. |
| b03. F - Other health-related benefits | Other mentions of taxation as helping improving health, including specific epidemiological data on diseases and deaths |
| b04. F- Addressing negative externalities | Taxation as addressing "negative externalities"<br><br>*Comment: in the context of the Peruvian study, the word "negative externality" was principally used by economists as a technical word in favor of taxation. And it was more used at a certain period of time.* |
| b06. F - Increase of labour productivity | Taxation would increase the labour productivity of the country, with the population being more healthy, thus able to work better |
| b07. F (but need more) - Low taxes | Taxes are still low and need to be increased |
| b08. F - Product reformulation or replacement | Taxation could promote product reformulation and the replacement of health-harming products for healthier options. |
| b.09 N - Inflation update | Taxation should be updated according to inflation. |
| b10. F - Following guidelines of multinational organizations | There is a need to follow standards and guidelines of international organizations such as the WHO or OECD. |
| b11. F - A source of funding for health campaigns and public policies | Taxation would help raise money for health campaigns and public policies. |
| b12. F/N - Economic impact | Taxation has a positive impact or at least will not have a negative impact on the economy. |
| b13. F - Comparison with other countries | Other countries have had positive experiences with health taxation. |
| b14. F - Counter-arguments | Data or evidence that refute opinions or other arguments that oppose health taxes. |
| b.15 F - Public opinion | Public opinion is in favor of the taxes (e.g., results of opinion polls showing support to the health taxes). |
| b16. F - Progressive tax increase | There is an need to progressively increase taxation for better health outcomes. |
| bz. F - Other | Other arguments in favour of taxation, that do not fit in the other codes.<br><br>*Comment: some arguments did not fit in other categories, but we felt they were important and did not want to loose sight of them. We considered the possibility of grouping them later in the analysis.* |
| c01. A - Unfair distribution | Criticism of health taxes because there are different taxation rates for similar products. |
| c02. A - Taxes are too high | Health taxes are too high. |
| c03. A - Proce increase / Sales decrease | Taxation increases the price of products or/and will cause a decrease in sales. |
| c04. A - Increase in smuggling | Taxation increases **smuggling** of the products being taxed. |

| **Codes (B =In favour; C =Against)** | **Definition** |
|---|---|
| c05. A - Increase in adulterated products | Taxation increases the **adulteration of products**. |
| c06. A - Promote the informal economy | Taxation promotes the informal economy, where **informal products** do not include a tax.<br><br>*Comment: we understood informality, in the context of this investigation, not necessarily as an illegal activity since it tolerated by the government in Peru* |
| c07. A - Increase in corruption | Taxation increases corruption. |
| c08. A - Volatility / unpredictable | Taxes are unpredictable or very volatile. |
| c09. A - Against market competition/favours market concentration | Taxation is against free market competition or/and will favor market concentration. |
| c10. A - Tax evasion | Taxation leads to more significant tax evasion. |
| c11. A - Decrease or loss of investments | Taxation causes a decrease in investments. |
| c12. A - Affect the economy | Taxation leads to negative economic growth or will be against economic recovery or similar arguments. |
| c13. A - Affects small businesses | Taxation has a greater impact on small businesses. |
| c14. A - People will choose cheaper and less healthy alternatives | Taxation leads to the consumption of cheaper alternatives that are harmful to health. |
| c15. A - Greater impact on low income households | Taxation has a greater economic impact on low-income households. |
| c16. A - Loss of employment | Taxation translates in losses of jobs. |
| c17. A - Counter-arguments | Data or evidence that refute opinions or other arguments that are in favour of health taxes. |
| c18. A - Should not have revenue purposes | Taxation should not have a revenue purpose. |
| c19.- Not enough or little debate. | There has been little or no discussion on health taxes. |
| C20. F/A/N - Lack of evaluation | Taxation must be evaluated after its implementation.<br><br>*Comment: This was specially mentioned in the context of the criticism of taxation.* |
| cz. A - Other | Other arguments in favour of taxation, that do not fit in the other codes. |

Supplementary material 4: Interview guide

| INTERVIEW GUIDE | |
|---|---|
| **Questions** | **Aim of question** |
| **Opening** | |
| *Could you tell us about your professional experience and how you have been involved with health taxes in your field?* | To know the role, function and/or position of the interviewee as well as his/her experience in the implementation of Excise Duties or how he/she has approached them. |
| *Are the answers you give in your personal name or are they on behalf of the institution/association you represent (more for public sector, industry)?* | |
| **Body** | |
| *How would you explain in one sentence and in your own words what health taxes are?* | Knowing their own definition as well as the words used to refer to the tax will help us to know how respondents understand health taxes, their framings and, therefore, their positions on health taxes. |
| *Do you consider that there have been positive effects of the implementation of health taxes in Peru? Which ones?* | To know if you consider that there have been positive effects of the implementation of health taxes and what they have been. |
| *Do you consider that there have been negative effects of the application of health taxes in Peru? Which ones? (ask about alternatives, solutions or improvements to the current application of the health taxes).* | To find out whether they consider that there have been negative effects of the implementation of health taxes and what they have been. |
| *When do you think that health taxes have been most discussed/debated at the public level (ask about 2016 and 2018)?* | To have an approximation of what the interviewee considers to be the most important moments in the implementation of health taxes and whether he/she considers 2016 or 2018 to be key moments. |
| *Taking into account your experience with health taxes, with which sectors or actors have you had the opportunity to collaborate or work together and in what way?* | Find out about their joint work with other institutions or associations in relation to health taxes. To have an approximation of what alliances they have generated. |
| *How do you evaluate the role of the state, and especially the MoEF (Ministry of Economy and Finance) through the last governments, with respect to the application of health taxes?* | To know if they identify a different emphasis in recent governments on the convenience or not of applying health taxes. |
| **Closure** | |
| *Who else would you recommend that we talk to about the topic of this research? Who do you consider to be the main actors for/against the implementation of health taxes in Peru (ask if you can contact any actors)?* | Find out if there are any relevant actors that could be considered for interviews that have not been considered before. |



Zuleta M, *et al. BMJ Glob Health* 2023; 8:e012024. doi: 10.1136/bmjgh-2023-012024

| | |
|---|---|
| *Are there any other aspects that have not been addressed that you would like to add more about?* | Give the interviewee the opportunity to add or incorporate any issue related to health taxes that he/she considers important and that has not been mentioned in the interview. |

Supplementary material 5: Quotations

| Cite Nº | Quotation | Source |
|---|---|---|
| 1 | *"We were in May 2016 (...) our authorities knew that they were not going to stay (due to the elections). So they said, well this is the time to do it. Because everyone is looking at the elections and they can't throw us out because we are leaving. So we basically increased the tax. It was a favourable context"* | MoEF official - interview |
| 2 | *During those years, there was also a lot of opposition, above all, a lot of lobbying, there was a lot of lobbying by the industries in the executive and legislative branches. In the legislative branch, the entire National Society of Industries, CONFIEP, etc., had their parliamentary coordinators, so they were more active and better organised than any parliamentary coordinator in any ministry, so there was no way to get anything out,* | civil society advocator - interview |
| 3 | *"And more or less in the year 2005-2006, this concept began to appear, it is already mentioned that selective consumption taxes (health taxes) are applied on goods and services that cause negative externalities, so the concept of negative externalities begins to appear in the guidelines"* | MoEF official - interview |
| 4 | *"Every time we have a modification, they come up with studies, with information, which in reality are not very reliable but which they can afford to finance and have public exposure of these reports. For example, in the case of tobacco, they point out that our tax increases have generated an illicit trade that is around 50% of what is consumed in Peru, for example. And this has appeared in all the media (...) But we have studies that GRADE* (think tank) *has done, independent studies, that nobody has financed, and they indicate that the illicit trade is less than 15% (...) In the case of alcoholic beverages the same thing happens, they say that the trade in alcoholic beverages in Peru is over 35% (...) but there are data that can easily disprove this information. What you have is that in Peru around 85% of the alcoholic beverages consumed is beer, and there is no illicit trade in beer. So, if 85% is beer, which is totally legal, at most, we would have a potential 15% of illicit beverages."* | MoEF official - interview |
| 5 | *the Association of Minimarket Salespersons has played an important role in the country (...) it has spoken out against several measures related to tobacco control (...). ) When the law (banning tobacco advertising, promotion and sponsorship) was proposed, the Peruvian Association of Winemakers played an important role, never before in the region had any association of winemakers spoken out against any measure, and it was the first time on the issue of tobacco control.... It has happened in Mexico, it has happened in other countries, but in Peru it was the first country where a Peruvian association of winemakers stood up in the Plaza Bolivar, in the square in front of the Congress, made a demonstration and had a huge presence in the media....* | civil society advocator - interview |
| 6 | *So there was a debate in Congress and if you listen to the representative of the Ministry of Economy, you would think that she is a person who represents health, I would say that she has presented better than any representative of the Ministry of Health". (Interview to civil society health tax advocator)* | civil society advocator - interview |
| 7 | *"My assessment is that it is a biased body" that "has been more dedicated to ideological issues than to practical ones".* | civil society representative against health taxes - interview |
| 8 | *"The MEF has good intentions, but it has a defect, it does not believe in the private sector, in other words, the private sector can stand on its head, and say hey, I am being affected by contraband and it does not believe it and in that sense it is stubborn and wrong, it should be a little more open or carry out its own study."* | public opinion leader against health taxes - interview |

| Cite Nº | Quotation | Source |
|---|---|---|
| 9 | *"Peru is moving in the right direction and establishing measures consistent with its tobacco control obligations,"* | PAHO representative - media |
| 10 | *"We (OECD representative for Latin America) see a country that has a relatively low tax collection, a country that collects around 16.1% of GDP, which is much lower than the 22.7% that Latin America collects and is less than half of what the OECD collects"* | OECD representative - media |
| 11 | *"Reducing the consumption of sugary and alcoholic beverages, avoiding smoking and discouraging the use of polluting vehicles are objectives with which it is easy to agree. Doubts arise, however, when it becomes known that the initiative for these increases comes from the Ministry of Economy and Finance (MEF) and not from the Ministry of Health (Minsa). So, is the objective to reduce consumption or to collect more taxes?"* | public opinion leader against health taxes - media |
| 12 | *"It (the law for advertising, promotion and sponsorship of tobacco) was scheduled for the last day (in the Health Comission of Congress), and when the law was going to be approved, the President of the Health Comission dissapeared, he left, called an intermission and never appeared again". (interview to civil society health taxes advocator).* | civil society advocator - interview |
